# STARD6 on steroids: solution structure, multiple timescale backbone dynamics and ligand binding mechanism

**DOI:** 10.1038/srep28486

**Published:** 2016-06-24

**Authors:** Danny Létourneau, Mikaël Bédard, Jérôme Cabana, Andrée Lefebvre, Jean-Guy LeHoux, Pierre Lavigne

**Affiliations:** 1 Département de Biochimie, Faculté de Médecine et des Sciences de la Santé, Université de Sherbrooke, Sherbrooke, Qc., Canada; 2 Institut de Pharmacologie de Sherbrooke, Université de Sherbrooke, Sherbrooke, Canada; 3 PROTEO; Regroupement Stratégique sur la Fonction, la Structure et l’Ingénierie des Protéines, Université Laval, Québec, Qc., Canada; 4 GRASP; Groupe de Recherche Axé sur la Structure des Protéines, McGill University, Montréal, Qc., Canada

## Abstract

START domain proteins are conserved α/β helix-grip fold that play a role in the non-vesicular and intracellular transport of lipids and sterols. The mechanism and conformational changes permitting the entry of the ligand into their buried binding sites is not well understood. Moreover, their functions and the identification of cognate ligands is still an active area of research. Here, we report the solution structure of STARD6 and the characterization of its backbone dynamics on multiple time-scales through ^15^N spin-relaxation and amide exchange studies. We reveal for the first time the presence of concerted fluctuations in the Ω_1_ loop and the C-terminal helix on the microsecond-millisecond time-scale that allows for the opening of the binding site and ligand entry. We also report that STARD6 binds specifically testosterone. Our work represents a milestone for the study of ligand binding mechanism by other START domains and the elucidation of the biological function of STARD6.

The StAR (Steroidogenic Acute Regulatory) protein is the archetype of a family of proteins having a START (STeroidogenic Acute Regulatory-related lipid Transfer) domain, a module of approximately 210 amino acids that adopts an α/β helix grip fold^1^,^2^,^3^. These proteins are expressed in plants and animals and are preserved through the evolution. In humans, there are fifteen different START domain proteins^4^, which are subdivided into six subfamilies: STARD1 (STARD1/D3), STARD4 (STARD4/D5/D6), STARD2 (STARD2/D7/D10/D11), STARD8 (STARD8/D12/D13), STARD14 (STAR14/D15) and STARD9^4^,^5^. These proteins bind a variety of lipids and sterols^6^ and have various patterns of expression and cellular localization. They are involved in the non-vesicular transport of lipids and sterols, their transfer, their metabolism and cell signalling^4^,^5^,^7^,^8^. Some STARTs have known ligands, but many have yet to have their cognate and functional ligands identified. However, members of the STARD1 and STARD4 subfamilies bind sterol molecules and we are reporting that STARD6 specifically binds C19 and C21 steroids^9^.

Whereas the three-dimensional structures of four of the five START domains of the STARD1 and STARD4 subfamilies; STARD1 and STARD5^10^, STARD3^2^ and STARD4^11^ have been solved, the structure of STARD6 remains to be determined. Moreover, the mechanism of ligand binding and dissociation is not exactly understood. Compared to classical cases of ligand binding, binding of sterol molecules (and other lipids) to START domains is complicated by the fact that the binding site is completely buried inside the α/β helix grip fold. As reviewed elsewhere^12^,^13^, different conformational changes have been proposed to give access and allow for the exit of ligands but their time-scales and synchrony remains to be determined. These structural changes involve the opening of the Ω_1_ loop, the C-terminal helix and concerted movements between the Ω_1_ loop and the C-terminal (α_4_) helix^2^ as well as between Ω_1_ loop and α_3_ helix^14^,^15^. The possible existence of an intermediate state with an open conformation involving local and reversible structural changes in the Ω_1_ loop and/or the C-terminal helix to allow for the entry of the ligand has also been suggested^16^. Identification and validation of the exact movements and their time-scales are of the utmost importance for our understanding of ligand binding by START domains and their functions as transporters of single lipid and sterol molecules.

In the present study, we report the NMR solution structure of apo-STARD6 and the dynamical characterization of its backbone in its free and testosterone bound forms on different time-scales using various ^15^N spin-relaxation NMR methods and amide exchange kinetics monitored by HSQC based methods. STARD6 adopts a typical α/β helix grip fold in solution and its backbone undergoes dynamical fluctuations on several time-scales. We observe fluctuations within the α_3_ and α_4_ helices, as well as in the Ω_1_ loop. Moreover, we demonstrate for the first time that these fluctuations occur on the picosecond-nanosecond (ps-ns), microsecond-millisecond (μs-ms) and longer time-scales. Unbiased molecular dynamic simulations on the μs time-scale have sampled structural rearrangements of STARD6 consistent with those fluctuations and suggest that they induce the coalescence of the internal cavity and the surrounding solvent, allowing for the ligand entry. This constitutes the first experimental demonstration of a detailed molecular mechanism for ligand binding by a START domain.

## Material and Methods

### Cloning, expression and purification

The cDNA for the human STARD6 was provided by Invitrogen (Clone ID 100015037), cloned in pET21b and sequenced. The construct contains a hexahistidine tag at the C-terminus. For ^15^N- or ^13^C, ^15^N-double labeling, ^15^N ammonium chloride (1 g/l) and ^13^C glucose (2 g/l) (Cambridge Isotopes) as the sole nitrogen and carbon sources were used. *E. coli* BL21(DE3) were transformed with the plasmid and grown at room temperature (23 °C) in M9 medium (100% H_2_O or 20% H_2_O: 80% D_2_O) and induced with 1 mM isopropyl β-D-1-thiogalactopyranoside (IPTG) when OD_600_ reached 0.6. Transformed *E. coli* BL21(DE3) were gradually selected to grow in D_2_O, up to 80%, prior to induction. After induction, cells were incubated for an additional 18 hours at room temperature prior to harvesting by centrifugation. Cells were then resuspended in lysis buffer (3 ml/g of pellets; buffer composition: 50 mM K-Phosphate, 500 mM KCl, 10 mM imidazole, 1 mM TCEP, pH7.4) with 1 mM TCEP, protease inhibitors (complete Mini EDTA-free inhibitors from Roche) and 1 mM PMSF and frozen at −80 °C.

Bacterial pellets were lysed by thawing at 37 °C followed by addition of lysozyme (1 mg/ml) and DNAse I (50 μg/ml). The cell lysate was then centrifuged at 19000 g for 30 min and the supernatant was loaded onto a Ni-NTA column (Qiagen) during 2 hours at room temperature. The resin was washed twice with lysis buffer and the STARD6 recombinant protein was eluted with elution buffer (50 mM K-Phosphate, 500 mM KCl, 250 mM imidazole, 1 mM TCEP, pH7.4). The protein was then exchanged and concentrated using Millipore UltraCel ultracentrifugation filters (10,000 Da MWCO; Amicon Canada) device into the NMR buffer (50 mM potassium phosphate, 50 mM KCl, 1 mM TCEP, pH 7.4) complemented with 10% D_2_O and 0,02% NaN_3_. The final concentrations of the NMR samples were between 0.8 and 1.1 mM. The purity of the final protein sample was confirmed by SDS–PAGE.

### CD spectropolarimetry and determination ligand binding affinity by thermal shift assay

Circular dichroism (CD) measurements were performed on a Jasco J-810 spectropolarimeter equipped with a Peltier-type thermostat. Experiments were performed using quartz cells with a path-length of 1.0 mm. For CD spectra and temperature-denaturation measurements of STARD6, the protein was dissolved in 10 mM sodium phosphate buffer at pH 7.4, to a final concentration of 6 μM. The protein concentration was determined spectrophotometrically at 280 nm using an extinction coefficient of 25900 M^−1 ^cm^−1^. The CD spectra presented are the results of the accumulation of ten scans at 0.1 nm intervals. Scan speeds and time constants were chosen to allow sufficient response time and achieve favourable signal-to-noise ratios. Temperature-induced denaturation curves were performed in the temperature range from 10 °C to 90 °C with a heating rate of 1 °C/min. The raw mdeg values were transformed in mean residue molar ellipticity (deg·cm^2^·dmol^−1^) using the following equation:


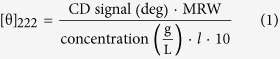


where MRW is the mean residue weight and *l* is the path length of the CD cell in cm. The apparent melting temperature (T°), apparent standard enthalpy of unfolding at T°, (ΔH°_u_(T°)) and apparent temperature dependent Gibbs free energy of unfolding (ΔG°_u_ (T°)) were determined by the simulation of the temperature denaturation curves with a model describing the equilibrium for a two-state unfolding mechanism as described in Roostaee *et al*.^17^. Measurements with the ligand were performed at protein to ligand molar ratios ranging from 1:0.5 to 1:5 and spectra were taken at a molar ratio of 1:5. For the binding studies, stock solutions were prepared in ethanol at a final concentration of ethanol of 0.4%. After 90 min equilibrium time, samples were analysed by CD. Each spectrum was baseline-corrected for buffer and ligand. The values of the apparent dissociation constant K_d_ (T) of STARD6-testosterone complexes were determined by the use of thermal shift analysis as described elsewhere^18^. Briefly, the increase in protein stability (or standard free energy of unfolding) due to ligand binding ΔΔG°_u,L_(T) is given by the difference in standard free energy of unfolding in the presence of the ligand (ΔG°_u,L_(T)) and the standard free energy of unfolding of the free protein ΔG°_u_(T). As demonstrated in detail by Layton and Hellinga, assuming that the ligand does not bind to the denatured state, the ΔΔG°_u,L_(T) is equivalent to the standard free energy of binding (ΔG°_b_(T)). In order to determine ΔΔG°_u,L_(T), the change in standard heat capacity of unfolding ΔC°_p,u,L_ must be estimated. This is done by estimating T° and ΔH°_u_(T°) for each ligand concentration and by plotting the resultant ΔH°_u_(T°) as a function of T°. ΔC°_p,u,L_ is assumed to be temperature independent and is given by the slope of the linear curve. Then ΔΔG°_u,L_ values are calculated at the temperature of the melting of the apo-state (T°_apo_) for various ligand concentrations and the affinity of the interaction is determined by fitting the canonical equation for the free energy of ligand binding in the stoichiometric regime:


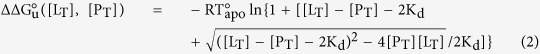


where [L_T_] and [P_T_] are the total concentration of the ligand and protein, respectively.

### NMR spectroscopy

All NMR experiments were performed at 298 K on a Varian 600 MHz spectrometer equipped with a Z-axis pulsed-field gradient triple resonance probes. The backbone sequence-specific assignments of ^1^HN, ^13^Cα, ^13^C’, ^15^N and ^1^Hα and side-chain ^13^C_β_ for the STARD6 were obtained using ^1^H-^15^N HSQC and the following standard triple-resonance NMR pulse sequences: CBCA(CO)NH, CBCANH, HNCO, HNCA, HNCACB, HN(CO)CA, CC(CO)NH and HNHA. Side-chains ^1^H and ^13^C chemical shifts were assigned using HCCONH, HCCH-TOCSY and CCCONH pulse sequences also included in the Biopack repertoire.

The chemical shift values of ^15^N, ^13^C_α_ and ^13^C_β_ have been corrected for the deuterium isotopic effect as described elsewhere^19^. ^15^N-Edited NOESY (mixing times of 50, 150 ms) and C^13^ (aliphatic and aromatic)-Edited NOESY (mixing time of 150 ms) were used to assign NOEs. NMR data were processed using NMRPipe^20^ and analyzed with CCPNmr Analysis^21^. Samples containing the ligand were prepared from an ethanolic solution of the steroid to a final ratio of 1: 1 and a final ethanol concentration of 2%.

### ^15^N Spin relaxation

^15^N-R_1_, R_2_ and {^1^H}-^15^N NOE experiments were recorded using previously described pulse sequences available in the Biopack repertoire^22^. Delays of 0, 30, 60, 90, 120, 150, 250, 350, 450, 550, 650, 800 and 1000 ms and of 0, 10, 30, 50, 70, 90, 110, 130, 150, 170, 250, 350 and 490 ms were used to obtain ^15^N-R_1_ and R_2_ respectively. {^1^H}-^15^N NOE measurements were done by comparing HSQC spectra with and without a 10-second proton saturation.

### Model free Analysis

In order to characterize the ps-ns time-scale backbone dynamics and explore the potential occurrence of conformational exchange (R_ex_) on the μs-ms time-scale, we have analysed the ^15^N relaxation data recorded as described above and using ModelFree 4.0^23^ in conjunction with Fast ModelFree^24^. Prior to proceeding to the ModelFree analysis, the center of mass of the STARD6 pdb structure was translated to coordinate origin using the program PDBINERTIA and the diffusion tensors for spherical and axially symmetric diffusion models were estimated on the basis of ^15^N-R_2_/R_1_ values for rigid amides with the program R2R1_DIFFUSION (AG Palmer: http://www.palmer.hs.columbia.edu/software/diffusion.html). S^2^ (squared order parameter of the NH bond vector), τ_e_ (internal correlation time) and R_ex_ (exchange broadening) were derived from the average of 2 ^15^N-R_1_, R_2_ and {^1^H}-^15^N NOE datasets (for both the apo- and holo-STARD6) using the common models of the spectral density function (*i.e.* S^2^, S^2^-τ_e_, S^2^-R_ex_, S^2^-τ_e_-R_ex_)^25^,^26^ and two-time-scale internal motion^27^. In both cases, the datasets were fitted best with an axially symmetric rotational diffusion tensor^28^. The axially symmetric rotational diffusion is defined by the anisotropy ratio, D_ratio_ = D_par_/D_perp_ where D_par_ is the rotational diffusion coefficient parallel to the unique axis of the diffusion tensor and D_perp_ is the diffusion coefficient perpendicular to it. In such a model, the local τ_m_ depends on the angle α, *i.e.* the angle between the NH bond vector and the unique axis of the diffusion tensor. The effective correlation time (τ_m,eff_) is given by 1/6D_iso_, where D_iso_ is equal to (2D_perp_+D_par_)/3.

### Relaxation Dispersion Data Analysis

The backbone amide ^15^N relaxation dispersion profiles were recorded by measuring their effective transverse relaxation rates, R_2¸eff_, with a constant time relaxation delay of 50 ms as a function of the refocusing CPMG field strengths, ν_cpmg_. The pulse sequence found in the Biopack repertoire which was derived from the work of Palmer *et al*.^29^ and Kay *et al*.^30^ was used. R_2¸eff_, with ν_cpmg_ ranging from 80 and 1000 Hz, were measured with a repeat at 480 Hz to estimate experimental error. Spectra were processed with NMRPipe^20^ and analysed with CCPNmr^21^. The effective transverse relaxation rates (R_2¸eff_ (ν_cpmg_)), comprising the transverse relaxation rate in the absence of exchange (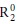), and the component arising from chemical exchange (R_ex_), were calculated via:


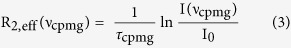


where I (ν_cpmg_) is the peak intensity at a given CPMG field strength and I_0_ is the peak intensity without the CPMG relaxation period. Relaxation dispersion curves were obtained by plotting R_2,eff_ as a function of ν_cpmg_. The data were fitted individually considering no-exchange (Eq. 4a) or using the all timescale approximation described by Ishima and Torchia (Eq. 4b)^31^ to identify residues affected by conformational exchange:


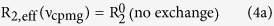


or


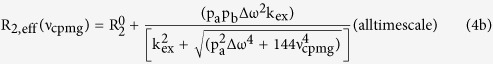


where R_2_^0^ is the exchange rate in absence of a conformational exchange contribution (R_ex_) or R_2¸eff_ (ν_cpmg_) when ν_cpmg_ tends to an infinite value, p_a_ and p_b_ are the populations of the two interchanging states where p_a_ ≫ p_b_ and p_b_ represent an intermediate and excited state. The Δω parameter is the difference in ^15^N chemical shifts in both states and k_ex_, the exchange rate, is given by k_A_+k_B_, the two rate constants of the exchange equilibrium. R_ex_ is given by:


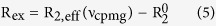


We then grouped residues for cluster analysis based initial k_ex_ values obtained, secondary structure elements and inter-residue secondary or tertiary connectivity. Parameters k_ex_ and p_b_ were shared parameters for each cluster, and R_2_^0^ and Δω were individually determined. Residues presenting crosspeak overlap were discarded from the relaxation dispersion analysis.

### Data processing and analysis

NMR data were processed using NMRPipe^20^ and analysed with CCPNmr Analysis^21^. The ^15^N-R_1_ and R_2_ rate constants and their uncertainties were obtained by fitting the decays of the peak heights to the classical two-parameter single exponential using the least squares fitting program in CCPNmr Analysis. The relaxation dispersion profiles were fit to equation 6 with the program GraphPad Prism 6 (http://www.graphpad.com/) for cluster analysis and to determine k_ex_, Δω and R_ex_ values and their uncertainties. The chemical shift displacements (CSD) were calculated with the following equation:


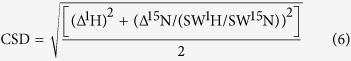


where SW^1^H and SW^15^N are the spectral widths in the ^1^H and ^15^N dimensions respectively. Significant displacements are ascribed to cross-peaks that have CSD values of one standard deviation (SD) above the mean.

### Structure calculations

All NOEs were assigned manually and converted into distance restraints using CCPNmr Analysis. The program DANGLE^32^ was used to obtain ϕ and ψ dihedral angles on the basis of backbone and ^13^C_β_ chemical shift values. The CLEANEX-PM pulse-sequence^33^ has been used to identify fast and slow exchanging backbone amides. H-Bond restraints have been used only for residues showing slow exchanging amides (not observed on the CLEANEX spectra and presenting high protection factors; see below) and found in regions of regular secondary structures. Structures were calculated using the program ARIA2.2^34^ in conjunction with CNS 1.21^35^. Of the 1000 conformers calculated, the 20 presenting the lowest-energy, the lowest number of NOE and dihedral violations were refined in water and submitted to PROCHECK_NMR^36^ to validate the structural quality. The final structure ensemble was deposited in the protein databank (PDB: http://www.rcsb.org/pdb/) under the identification code 2MOU.

### Amide proton exchange experiments

Slow exchange of amide protons was monitored by standard hydrogen–deuterium (H/D) exchange experiments, in which H/D exchange was initiated using the quick dissolution of lyophilized sample techniques at 298 K. A series of ^1^H-^15^N HSQC spectra were acquired after hydration of the lyophilized samples in D_2_O at various time intervals up to 4 days. Peak intensities were integrated with CCPNmr Analysis. To obtain k_ex,HX_ values, a single exponential function was fitted to the time-dependent decay of signal intensities of non-overlapping cross-peaks:


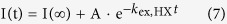


where I(t) is the signal intensity at time *t*, I(∞) is the signal intensity at infinite time, A is the maximum intensity change for a given signal, given by I(t) at *t* = 0 minus I(∞), and k_ex,HX_ is the site-specific observed rate constant for exchange. Fast exchange of amide protons were identified using the CLEANEX-PM pulse sequence^37^. Each spectrum was recorded with various mixing times (0, 5, 10, 20, 40, 60, 80, 100, 150 and 200 ms). The rates of exchange (k_ex,CLX_) were determined from fitting the CLEANEX hydrogen exchange build-up curves using Equation 6 with GraphPad Prism 6 (http://www.graphpad.com/); where τ_m_ is the mixing times, I the corresponding peak intensity and I_0_ is the reference peak intensity (τ_m_ = 0)^33^.


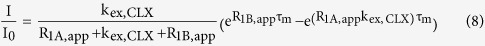


The previously determined value of 0.6 s^−1^ was used for the apparent relaxation rate of water (R_1B,app_). Amide proton protection factors (P_f_) were calculated from the results of all hydrogen exchange experiments as k_int_/k_ex,CLX_ or k_exHX_, where k_int_ are the intrinsic exchange rate constants that were estimated using the program SPHERE, based on methods developed by Englander and co-workers (http://landing.foxchase.org/research/labs/roder/sphere/sphere.html)^38^.

### Molecular dynamics simulations of apo-STARD6 and its docked complex with testosterone

The GROMACS software suite^39^,^40^ was used to prepare and run the simulations. The STARD6 protein and its docked complex with testosterone was soaked with the SPC water model^41^ and approximately 150 mM NaCl, keeping the net charge of the system at zero. The STARD6 protein was centred in a dodecahedric box in periodic boundary conditions with each side of the box being at least 10 Å from the protein to prevent any interaction between the periodic images of STARD6. The Gromos 54a7 force field was used for the calculations^42^,^43^. The testosterone molecule and parameters were obtained on the Automated Topology Builder repository (Molecule ID 9924)^44^. The testosterone molecule was docked in the binding site of STARD6 using FlexAID^45^ as described in Letourneau *et al*.^46^. Equilibration of the system under conditions of constant number of molecules, volume and temperature (NVT) was performed for 10 ps to reach the desired temperature of 310 K. This was followed by equilibration under conditions of constant number of molecules, pressure and temperature (NPT) for 100 ps with the pressure set at 1 bar. Both these steps were performed with position restraints on the heavy atoms of the protein and ligand. A second step of 100 ps of equilibration in NPT condition was performed with position restraint applied only to the backbone atoms of the protein. Unrestrained MD simulations were run in 2 fs steps for 5 μs of total MD simulation time, in the form of 10 simulations of 500 ns in length for each simulated system. The simulations were run in periodic boundary conditions at constant temperature (310 K) and pressure (1 bar) using the Nose-Hoover thermostat^47^,^48^ with τ_T_ = 0.5 ps and the Parrinello-Rahman barostat with τ_P_ = 2 ps, respectively. Simulated frames were saved every 20 ps, for a total of 250 000 frames per 5 μs simulation. MD trajectories output from GROMACS were converted to PDB files for visual inspection with PyMOL (https://www.pymol.org/) and to compressed XTC trajectory files for other analyses. The root mean squared fluctuation (RMSF, a measure of the average backbone mobility over time) analysis was performed using the *g_rmsf* tool within GROMACS.

## Results and Discussion

### Apo-STARD6 thermodynamical stability

**Figure 1:**
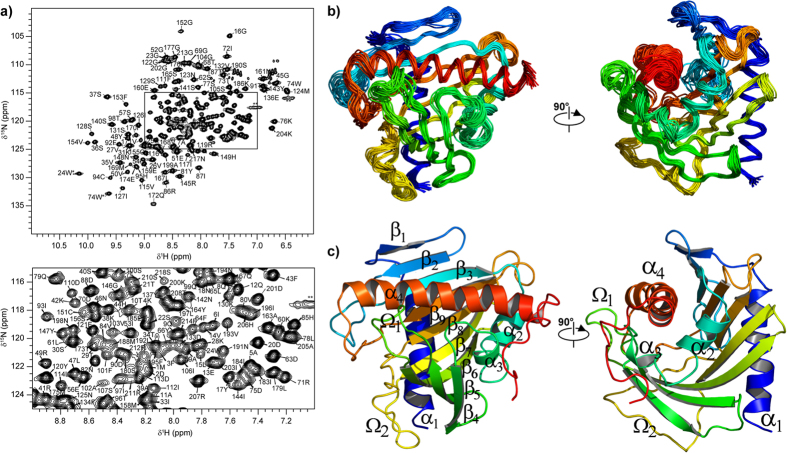
Solution structure of STARD6 (2MOU). (**a**) ^1^H-^15^N-HSQC spectrum of STARD6 (top). Assignments of the cross-peaks are given with the residue number and one-letter code for amino acids. Tryptophan side-chain indole NH cross-peaks are marked with an asterisk (*). The double asterisks (**) denote protected guanidino (Hη) protons from arginine side-chains. Detailed view of the more congested central region of the spectrum (bottom). (**b**) Backbone overlays of the 20 lowest energy structures ensemble. The last 10 residues of the C-teminal were omitted for the sake of clarity. (**c**) Cartoon representation of the lowest energy structure. Secondary structure elements are labelled. The protein model is rainbow-colored, from the N-terminus in blue to the C-terminus in red.

**Figure 2:**
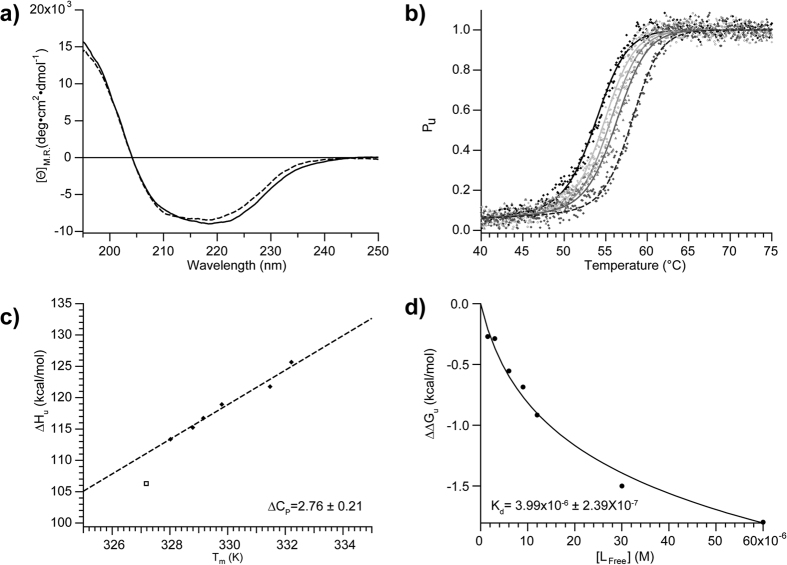
Far–UV CD spectroscopy of STARD6, experimental determination of ΔC°_p,u,L_ and apparent K_d_ value for STARD6-testosterone complex. (**a**) Mean residue ellipticity of STARD6 (full line) and in complex with testosterone (dashed line). Each spectrum was baseline-corrected for buffer and ligand. (**b**) Thermal melting curve recorded at 222 nm and temperature–dependence of the population of unfolded state (P_u_) for Apo-STARD6 (full line) and in complex at a ratio of 1:2 (light grey), 1:1 (mid grey), 2:1 (dark grey) and 5:1 (dashed line) with testosterone. (**c**) Estimation ΔC°_p,u,L_ by a linear fit of ΔH°_u_(T°) *vs.* T° values determined experimentally. The value for the apo-STARD6 (◻) falls under the fit line, reflecting the absence of the ΔH°_b_ contribution to ΔH°_u_. (**d**) Dependence of ΔΔG°_u,L_(T) on testosterone concentration that can be fit for a K_d_ value at the T° of the apo-state using the model in which ligands binds selectively to the native state. The concentration of proteins is 6 μM in 10 mM sodium phosphate buffer at pH 7.4. Ligands were dissolved in ethanol were used and the final concentration of ethanol was 0.4%. Samples were analysed after an equilibration period of 90 min.

The ^1^H-^15^N-HSQC spectrum of STARD6 is well resolved with a single set of resonances (Fig. 1a). This indicates that STARD6 has a stably folded tertiary structure. Accordingly, the thermal denaturation monitored by CD (Fig. 2a,b) fits a two-state model (equilibrium between a folded and unfolded state) and van’t Hoff analysis yields a T° and a 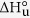
(T°) of unfolding of 54.0 ± 0.3 °C and 106.5 ± 2.3 kcal/mol respectively (Table 1). From these parameters and the Gibbs-Helmholtz equation, the free energy change of denaturation (ΔG_u_°(T)) at 25 °C and 37 °C are 9.4 ± 0.5 and 4.6 ± 0.5 kcal∙mol^−1^ respectively (Table 1), indicating that the population of native state of STARD6 is virtually 100% at both temperature. Overall, the data presented in this section suggest that STARD6 is stably folded and that if an intermediate state is present, it has a very low population and it is not in slow exchange with the ground state on the chemical shift time-scale.

### The solution structure of apo-STARD6

The solution structure of apo-STARD6 was determined by NMR spectroscopy using established methods for the assignments of the main chain and side-chain ^1^H, ^13^C and ^15^N chemical shifts and NOE. Amide exchange was used to identify fast and slow exchanging H^N^ atoms and infer their involvement in stable H-Bonds. Overall, 97% of the backbone and 93% of the side-chain atoms (^1^H, ^13^C and ^15^N) were assigned leading to 5103 NOE constraints, 302 dihedral angles and 80 H-bonds restraints. The detailed statistics can be found on the BioMagResBank (http://www.bmrb.wisc.edu/) with the accession number 18777/19952 and in Table 2.

**Figure 3:**
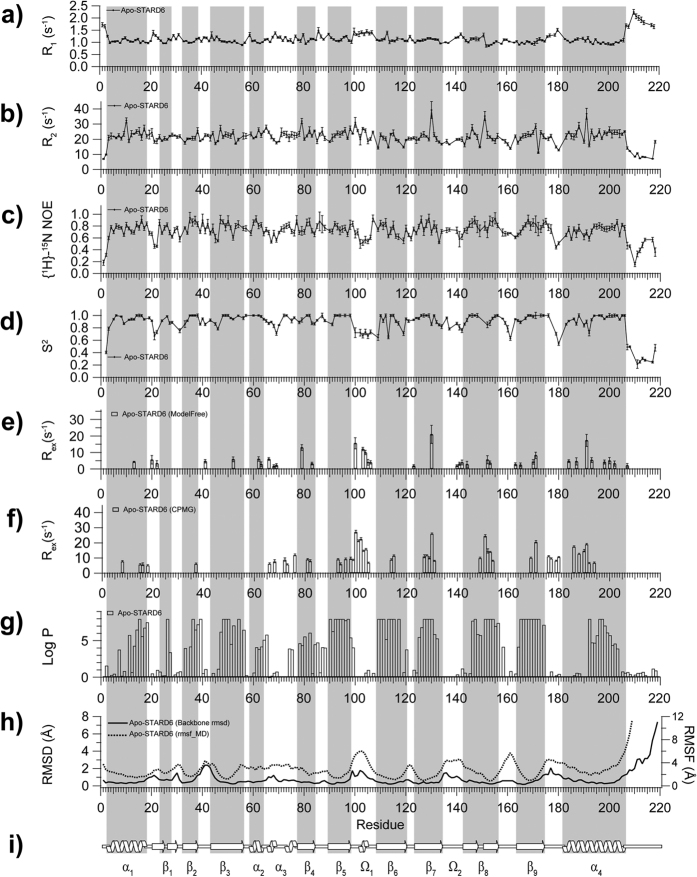
Backbone dynamics of apo-STARD6. ^15^N relaxation parameters at 600 MHz; (**a**) R_1_, (**b**) R_2_ and (**c**) {^1^H}-^15^N heteronuclear NOE. (**d**) Squared order parameter S^2^ and (**e**) R_ex_ parameters contribution derived from Model-free analysis. (**f**) Plot of the R_ex_ parameters obtained from the anaysis of relaxation dispersion curves. (**g**) H^N^ protection factors obtained from CLEANEX or standard H/D experiments. (**h**) Backbone mean RMSD from the 20 lowest energy structures ensemble (black line) and backbone RMSF plot from the MD simulation (dashed line) (**i**) Secondary structure profile from CSI values for the apo-STARD6.

The 20 lowest-energy structures [PDB ID code 2MOU] are shown superimposed onto the geometric average in Fig. 1b. The structure statistics for the entire protein, including the flexible ends, are summarized in Table 2. The averaged pairwise root-mean-square deviation (RMSD) calculated for backbone-only and for all heavy atoms are 1.18 (±0.18) and 1.8 (±0.14) Å, respectively. Those for the START consensus sequence (residues 1–208) are 0.8 (±0.10) and 1.39 (±0.12) Å and those for the regular secondary structure (α and β) are 0.43 (±0.07) and 0.98 (±0.10) Å, respectively. Close inspection of Figs 1b and 3h and Supplementary Fig. 1a indicates that the most structurally variable region are located in loops and encompasses residues 38–44, 99–107, 135–142, 174–182 and the C-terminal end of α_4_ helix beyond residue 206. Shown in Fig. 1c is a cartoon diagram of the most representative conformer of the ensemble based on its lowest potential energy. Collectively, those results confirm that STARD6 adopt a stable 3D structure consisting of the typical START domain topology.

### ^15^N-relaxation and picosecond-nanosecond backbone dynamics of apo-STARD6

^15^N-R_1_ and R_2_ and {^1^H}–^15^N heteronuclear Nuclear Overhauser Enhancement (NOE) were measured at 600 MHz (Fig. 3a–c) and used to characterize the picosecond-nanosecond (ps-ns) dynamics of the backbone of apo-STARD6. The average R_1_, R_2_ and NOE at at 600 MHz are 1.14 (±0.21) s^−1^, 21.3 (±4.7) s^−1^ and 0.72 (±0.13), respectively. Higher R_1_ and lower NOE and R_2_ than the average are observed in the loop between α_1_ and β_1_, the loop extending to the N-terminal strand of β_3_, the short α_3_ helix and the beginning of β_4_, the Ω_1_ and Ω_2_ loop, the loops between β_6_/β_7_ and β_8_/β_9_, the loop before α_4_, as well as the N- and C-termini of the protein (Fig. 3a–c). In accordance with the backbone RMSD (Fig. 3h and Supplementary Fig. 1a), this is indicative of internal motion with significant amplitude on the ps-ns time-scale. On the other hand, R_2_ values in the Ω_1_ loop (98–105), structurally juxtaposed to strands β_7_, β_8_ and the C-terminus of β_9_, as well as the N-terminal part of α_4_ helix, shows larger values than the average, indicative of conformational exchange on the μs-ms time-scale and/or rotational diffusion anisotropy (Fig. 3b).

To further characterize fast internal motions and conformational exchange on the backbone of STARD6 and to evaluate the existence of rotational diffusion anisotropy, we have analysed the ^15^N-relaxation parameters using the model-free approach as described in the methods section. The best statistics for the simulation of the datasets were obtained using an axially symmetric diffusion tensor with a D_ratio_ of 1.15 and a τ_m,eff_ of 14.5 ns indicating the existence of sizeable rotational diffusion anisotropy. The τ_m,eff_ estimated is in agreement with the correlation time reported for monomeric protein of similar molecular weight. Accordingly, the agreement between the locations of the R_ex_ derived from the model-free analysis and those determined from the relaxation dispersion curves was found to be optimal when the axially symmetric diffusion tensor was used (see below). The structure of STARD6 in the diffusion frame, depicting the unique axis (aligned with the α_4_ helix and perpendicular to the α_1_ helix) of the diffusion tensor, is presented in Supplementary Fig. 2a. Also shown in Supplementary Fig. 2b, are the derived angles (α) between the NH vectors and the unique axis of the diffusion tensor. In absence of conformational exchange (R_ex_ = 0) and internal motions (S^2^ = 1), NH vectors with α angle aligned with the unique axis (0 or 180°) will possess the longest local τ_m_ and highest R_2_. On the opposite, NH vectors with α angle around 90° will possess the shortest local τ_m_ and lowest R_2_. For a D_iso_ of 1.15 and τ_m,eff_ of 14.5 ns, these R_2_ values are 21.2 s^−1^ and 19.7 s^−1^, respectively. This difference represents the largest effect expected of rotational diffusion anisotropy on R_2_ and hence cannot explain the extreme (high) values observed in the Ω_1_ loop, strands β_7_, β_8_, the C-terminus of β_9_ and N-terminal part of α_4_ and strongly suggest the existence of conformational exchange in these regions of STARD6.

We report, in Fig. 3d,e, the S^2^ and the R_ex_ values that best fit the dataset as a function of the residue number according to the model free analysis. As can be observed, the derived S^2^ indicate relatively small amplitude motions (average S^2^ of 0.88 ± 0.10 for non-overlapping cross peaks) for the backbone across the protein with moderate fluctuation (0.8 < S^2^ > 0.5) localized in the loops between secondary structure elements (Supplementary Fig. 1b). As could be expected from end fraying effects, more pronounced movements are observed at both terminal ends. Excluding these (and considering only residues 5–207), on average, STARD6 has an S^2^ value of 0.91 (±0.11), which is significantly higher than the average S^2^ (0.81 ± 0.10) of residues in the loops. As one could expect, this indicates that the backbone in the loops is significantly more mobile than those of the core structure on the ps-ns time-scale. More interestingly, the model-free analysis yielded scattered R_ex_ with some contiguous values, especially in the Ω_1_ loop and the C-terminal helix where elevated R_2_ were observed (Fig. 3e). This suggests that those sections of STARD6 are sensing global motions on the μs-ms timescale. It is worth mentioning that such motions have been previously shown to be important for the regulation of the activity of different proteins 49,50,51 and thus, we decided to further characterize them using other approaches.

### μs-ms dynamics of Apo-STARD6

**Figure 4:**
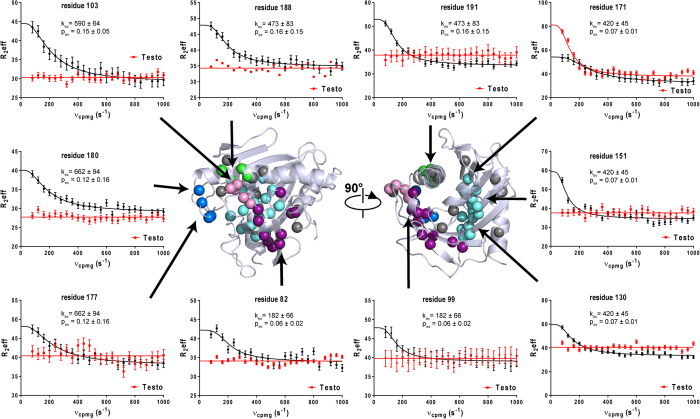
Microsecond-to-millisecond protein dynamics of apo-STARD6 and in complex with testosterone studied relaxation dispersion at 600 MHz. Representative dispersion profiles for each cluster of residues displaying R_ex_ values in the apo-STARD6 form (black curves) and in the presence of ligand (red) are indicated by coloured spheres on the structure. Population (P_b_) and k_ex_ values are also displayed. See text for detail. Most residues have their motions dampened except residues 171.

To confirm the presence and the identity of the backbone amide ^15^N undergoing or sensing conformational exchange, we performed a CPMG relaxation dispersion analysis. Representative relaxation dispersion curves are displayed on Fig. 4 and Supplementary Fig. 3. The majority of the residues presenting a R_ex_ in the model-free analysis are also found to be affected by conformational exchange by this technique. Moreover, the analysis of relaxation dispersion curves has led to the detection of a region (176–180) experiencing conformational exchange that was not revealed by the model-free analysis (Fig. 3e,f and Supplementary Fig. 1c). More specifically, these residues are located in the α_3_ helix, the C-terminal end of β_4_ (81–82) and β_5_ (93–98) strands, the Ω_1_ loop (99–105) and the N-terminal end of α_4_ helix (186–194) (Fig. 3f and Supplementary Fig. 1c). In addition, some residues of the β-sheet, for which the side-chains point toward the binding cavity (notably, residues 114–115, 127–131, 149–154 and 169–171), are experiencing conformational exchange (Fig. 3f and Supplementary Fig. 1c). We display on Fig. 4 and Supplementary Fig. 4a,b, the result of a cluster analysis of the dispersion curves. As can be observed, the contiguous residues at the right side of the β-sheet (Fig. 4: cyan spheres), could be fitted with a P_b_ of 0.07 ± 0.01 and a k_ex_ of 420 ± 45 s^−1^. Within statistical error, we could cluster another set of amides with similar k_ex_ and P_b_ and that belong to residues in the central part of the α_4_ helix (green spheres). A cluster with lower apparent k_ex_ and P_b_ involving residues in the α_3_ helix and strands at the left edges of the β-sheet was also detected (purple spheres). Comparatively, high B-factors were also observed for the α_3_ helix in the crystal structure of other START domain^52^. Finally, two other clusters of residues in the Ω_1_ loop (pink spheres) and in the preceding loop of the N-terminal part of the α_4_ helix with comparable k_ex_ (590 ± 64 s^−1^, 662 ± 94 s^−1^, respectively) and P_b_ (0.15 ± 0.05 and 0.12 ± 0.16) values were identified. Residues that we were unable to cluster, based on initial k_ex_ approximation and location, were not included in the cluster analysis (grey spheres) (Fig. 4 and Supplementary Fig. 4a,b).

### Beyond ms and slower dynamics of the apo-STARD6 form

In order to complete the characterization of the time-scale and amplitude of the conformational fluctuations present on the backbone of apo-STARD6, we have monitored the H^N^ exchange rates of the backbone amides at 25 °C. The rates of exchange (k_ex,CLX_) of fast exchanging amides were measured by the phase-modulated clean chemical exchange pulse scheme. The k_ex,HX_ of slow exchanging H^N^ were measured by the standard hydrogen–deuterium (H/D) exchange experiments, where the exchange is initiated by the quick dissolution of lyophilized sample in D_2_O and monitored by recording ^1^H-^15^N HSQC as a function of time. Protection factors (P_f_ = k_int_/k_ex,CLX_ or k_ex,HX_) were determined as described in materials and methods and are presented on Fig. 3g. Assuming that the exchange rates measured obey to the EX_2_ regime, *i.e.* that the closing rate of the opened structure from which the exchange takes places is larger than its intrinsic rate of exchange (k_int_), k_obs_ depends on the equilibrium constant of the determinant opening reaction (K_op_(T) = e^−∆Gop(T)/RT)^) and is given by P_f_^−1^. More than 30% of the total residues have completely exchanged with deuterium within the experimental dead time (30 min). These residues are found to be located in the loops and regions of α_3_ and the N-terminal of α_4_ as well as on the exposed edges of β-strands (Fig. 3g,i and Supplementary Fig. 1d). The central β-sheet structure and parts of helices α_1_ and α_4_ contain the most protected amides of the protein. The volumes of those cross-peaks have not significantly decreased even after four days. These amides have logP_f_ values over 8. This corresponds to ΔG_op_ values equal or larger than 10.5 kcal/mol and a population (P_op_(T) = e^−∆Gop(T)/RT^/(1 + e^−∆Gop(T)/RT^) of the opened state equal to or smaller than 10^−8^. This value is in agreement with the 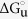
(25 °C) determined from the temperature denaturation curve for two-state unfolding. As seen on Supplementary Fig. 1d, these residues constitute a central and ring-shaped hydrophobic core around the cavity. Intermediate values of log P_f_ between 3.5–7.5 are observed for contiguous regions. Of interest, helices α_2_ and α_3_, strand β_4_ and the C-terminal part of helix α_4_, which make tertiary contacts, have log P_f_ values near four. Such a protection factor indicates that this portion of the tertiary structure of STARD6 has a smaller and independent ΔG_op_ of around 5.4 kcal/mol and a population of the opened state of 10^−4^. Similar local ΔG_op_ for amides at the interface of the α_4_ helix and strands β_1_ and β_2_ were also observed. As could be expected, amides exposed to the solvent and not potentially involved in H-bonds or more remote from the central core have the fastest exchange rates. At best, they have protection factors with log values around 0.5. Such values correspond to ΔG_op_ close to 1 kcal/mol and a population of the opened state that can reach up to 0.5. Particularly, one can notice that such weakly protected amides are located in the N-terminal part of the α_4_ helix and the Ω_1_ loop (Fig. 3g–i and Supplementary Fig. 1d).

### 5 μs Molecular Dynamics Simulation of Apo-STARD6

In an effort to further explore and rationalize the structural fluctuations detected on all time-scales, we have run a molecular dynamics simulation as described in details in material and methods. Briefly, the energy minimized average structure was soaked in a box of water and its cavity was filled with water molecules. This system was equilibrated for 210 ps before the simulation was started. We present on Fig. 3h, the RMSF calculated for the ensemble of frames collected over a total simulation time of 5 μs. One can notice a general agreement between the RMSD and the RMSF with the lowest RMSF values observed in the central core residues and the highest observed for residues distant from the central core. However, compared to the RMSD, the larger RMSF values indicate the presence of fluctuations larger than those observed on the ns-ps time-scale. In accordance, inspection of the trajectories revealed that, in accordance with its low P_f_ and concomitant to local fluctuations in the tertiary contacts involving the Ω_1_, that the N-terminal part of the α_4_ helix undergoes local denaturation. Collectively, these large amplitude motions lead to the coalescence of the internal water molecules (and cavity) with the surrounding solvent, suggesting a role for these global motions in the regulation of ligand entry in the binding cavity.

### Binding of testosterone to STARD6

In a recent analysis of the volumes and the shapes of the cavities and ligands of the START domains from the STARD4 subfamily, we had noticed that the cavity of STARD6 was significantly smaller, suggesting that smaller sterols such as steroids might be potential ligands for STARD6^9^. We demonstrate below that STARD6 can bind steroids with high affinity using testosterone as an example. First, we have used a circular dichroism-based thermal shift assays (see materials and methods) to determine the affinity of testosterone for STARD6 (Fig. 2 and Table 1). As for STARD5^46^,^53^, no noticeable changes in the spectrum and in secondary structure content are observed for STARD6 in the presence of ligand. However, the thermodynamical stability of STARD6 is significantly increased in the presence of increasing amounts of testosterone as expected for favourable binding of a ligand (Fig. 2b). The quantitative analysis of a two-state equilibrium model from thermal denaturation linked to binding (see materials and methods and Table 1) provided a K_d_ values of 3.08 × 10^−6^ at 54 °C (Fig. 2c,d and Table 1).

### The binding site of STARD6

**Figure 5:**
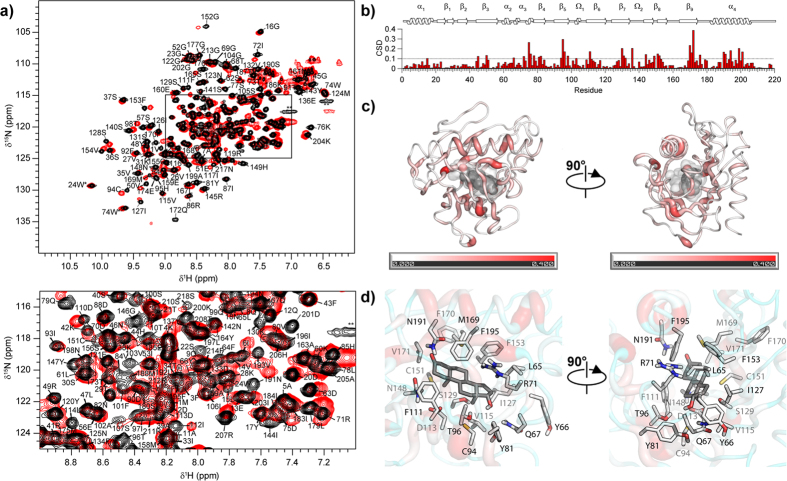
The Binding site of STARD6. (**a**) (top) Overlain of ^1^H-^15^N-HSQC spectra of apo-STARD6 (black) and in the presence of testosterone (red). Detailed view of the more congested central region of the spectra (bottom). (**b**) Weighted chemical shift displacements (CSD) observed upon testosterone binding to STARD6. Dashed line represented the mean value of CSDs. Significant displacements are assigned to cross-peaks that have CSD values one standard deviation above the mean (dotted lines). (**c**) Normalized CSDs mapped onto STARD6 structure for testosterone. Worm representation with worm radius proportional to CSD values and coded in a cyan-to-red gradient; regions without a significant perturbation (cyan) have a thinner backbone worm, regions of higher perturbations (red) have a thicker backbone worm, and intermediate regions are white. (**d**) Molecular docking of the STARD6-testosterone complex in accordance with the CSD data.

**Figure 6:**
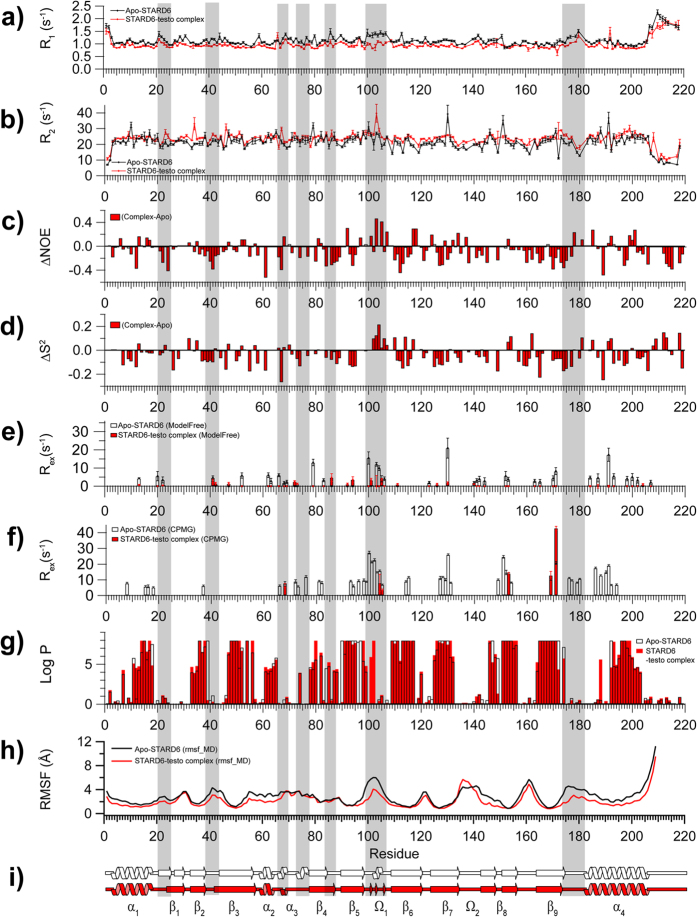
Backbone dynamics changes upon testosterone binding to STARD6. ^15^N relaxation parameters measured at 600 MHz; (**a**) R_1_ and (**b**) R_2_ and (**c**) {^1^H}-^15^N heteronuclear NOE difference (testosterone-apo). (**d**) Difference (testosterone-apo) in squared order parameters S^2^. (**e**) Plots of the R_ex_ parameters determined form the Model-free analyses for apo-STARD6 (open bar) and STARD6-testosterone complexes (red bar). (**f**) Plot of R_ex_ parameters determined from the analysis of the relaxation dispersion profiles (open bar) and STARD6-testosterone complexes (red bar). (**g**) H^N^ exchange protection factors from CLEANEX or standard H/D experiments for apo-STARD6 (open bar) and STARD6-testosterone complexes (red bar). (**h**) Backbone RMSF plot obtained from the MD simulation for apo-STARD6 (black line) and STARD6-testosterone complex (red line) (**i**) Secondary structure profile from CSI values for apo-STARD6 (white) and STARD6-testosterone complexes (red).

The binding site of STARD6 was defined by the classical SAR by NMR approach^54^. All the chemical shifts of the backbone of STARD6 were reassigned upon testosterone binding (BMRB accession number 26763) and the chemical shifts displacements (CSD) of all the backbone amide cross-peaks caused by the presence of the ligand were measured. Numerous cross-peaks on the ^1^H-^15^N HSQC of STARD6 are perturbed in presence of testosterone, indicating that their chemical environment is modified following ligand binding (Fig. 5a). As observed for the binding of Cholesterol to STARD1^12^,^13^ and bile acids to STARD5^46^,^53^, the binding of testosterone to STARD6 is in the slow exchange regime (not shown). In agreement with CD measurements (Fig. 2a), apart from helix α_3_ and the Ω_1_ loop, testosterone did not alter significantly the secondary structure of apo-STARD6 (Fig. 2a, Fig. 6i and Supplementary Fig. 5). The secondary chemical shifts for the Cα, Cβ, C’ and Hα as well as the CSI relative to the primary and secondary structures for the apo-STARD6 and STARD6-testosterone complex are shown in Supplementary Fig. 5. From the inspection of the CSDs (Fig. 5b) and the worm representation (Fig. 5c), the most perturbed amides are those that belong to the residues surrounding the cavity or that are next to it (i.e. 72, 75–76, (α_3_), 78, 81 (β_4_), 94–96, 98 (β_5_), 111 (β_6_), 130–132 (β_7_), 151–153 (β_8_), 170–172 (β_9_), 190–191, 194 and 199 (α_4_)); clearly demonstrating that the testosterone is located inside the cavity. Some remote residues (i.e. 45, 50, 82, 103, 136, 144, 149, 176 and 201) have their chemical shifts also perturbed by the presence of ligand, most likely originating from differential interactions with residues perturbed by the direct interaction with the ligand.

Based on CSD and on the shape of the cavity we have generated a complex between STARD6 and testosterone using molecular docking (Fig. 5c). Inspection of the complex reveals that the central part of testosterone is involved in non-polar interactions involving (C94, F111, V115, I127, C151, F153, M169, F170, V171 and F195) (Fig. 5d). In addition, testosterone possesses two polar groups capable of accepting and donating hydrogen bonds: the carbonyl (C=O) at C3 and a hydroxyl (OH) at C17. Interestingly, residues near both these polar moieties are polar in nature (Q67, R71, S129, N148 and N191) and can engage interaction to fulfill H-Bond potential. Altogether, these observations support the possibility that this configuration of the complex could be stable and highly representative.

### Changes in the backbone dynamics of STARD6 in complex with testosterone

^15^N relaxation parameters of testosterone-bound STARD6 were best fitted with a D_ratio_ of 1.15 and a τ_m,eff_ of 18.5 ns. This is a significant change compared to the τ_m,eff_ of 14.5 ns of the apo-form. Such a reduction in molecular tumbling is manifested by the general decrease in R_1_ and increase in R_2_ (Fig. 6a,b). Such an effect can be caused by one or a combination of the following phenomena: i) a conformational changes, ii) aggregation or iii) increase of the solvent viscosity. Since no significant change in the secondary chemical shifts of the backbone atoms between the apo- and holo-states have been observed, the possibility of a conformational change appears unlikely. Moreover, the CSD (*vide supra*) observed upon the addition of testosterone are localized around the binding site and do not define any other contiguous surface that could define a specific oligomerization interface. Hence, we trust that this increase in τ_m,eff_ is a result of either no-specific and weak aggregation and/or an increase in viscosity due to a slight excess of testosterone.

**Figure 7:**
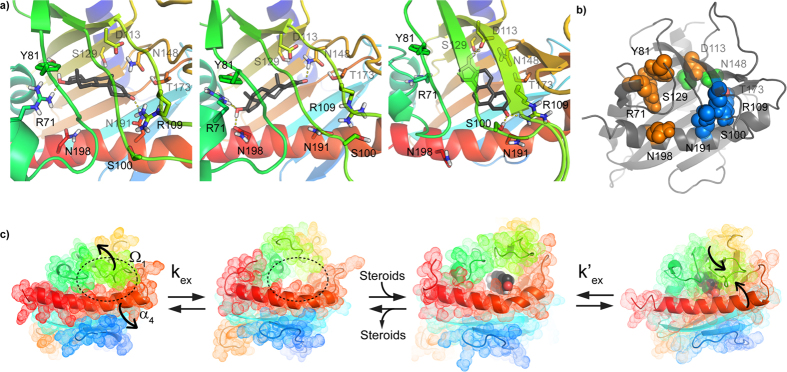
Binding mechanism and molecular dynamics simulation. (**a**) Representative snapshots taken from trajectories of the molecular dynamics simulation of STARD6-testosterone complex in which the ligand position changes while maintaining H-Bonds and non-polar interactions. Carbon atoms of the STARD6 model are rainbow-colored, from the N-terminus in blue to the C-terminus in red. Carbon atoms of testosterone are colored black. Oxygen, nitrogen and hydrogen atoms are respectively red, blue and white. The backbone is shown in cartoon representation and side chains are shown as sticks. H-bonds are shown as yellow dash. (**b**) The main polar interactions between the testosterone hydroxyl group and STARD6 occurs via R71, Y81, D113, N198 (orange) and S129 (green), whereas the testosterone carbonyl group interacts mainly through S100, R109, T173, N191 (blue) and N148 (green) or S129. (**c**) STARD6 is in equilibrium (k_ex_) with an excited state in which fluctuations (cartoon arrows) of the Ω_1_ loop and the N-terminal portion of the α_4_ helix induces the coalescence of the internal cavity of apo-STARD6 with the external solvent. Dashed line defines the internal cavity of apo-STARD6 fusing with the solvent. Upon steroids binding, a new equilibrium (k’_ex_) with a more frequent closed state appears. The backbone is shown in cartoon representation and semi-transparent spheres represent the molecular surface of the protein. Testosterone is shown in opaque spheres representation. Colors are the same as in (**a)**.

More interestingly, we observed a general decrease of S^2^ and {^1^H}-^15^N NOE in the backbone of the bound state of STARD6 compared to its apo-form. The average values are 0.88 (±0.16) (apo) *vs.* 0.82 (±0.15) (holo) for S^2^ and 0.72 (±0.13) (apo) *vs.* 0.65 (±0.21) (holo) for {^1^H}-^15^N NOE. More specifically, these gains of flexibility are located in the β_2_/β_3_ loop (36–41), the α_3_ helix (66–78), the β_4_ strand (83–88), the β_5_ strand (90–95), the β_6_ strand (111–116), the β_7_ strand (125–132), the Ω_2_ loop (135–142), the end of β_8_ and into the following loop (150–163), the C-term of β_9_ (170–177) and the α_4_ helix (189–206). However, an increase of rigidity on this time-scale occurs in the Ω_1_ loop region upon ligand binding as denoted by the difference (testosterone-apo) of squared order parameter S^2^ and {^1^H}-^15^N NOE values (Fig. 6c,d and Supplementary Fig. 7a,b). Thus, beside the Ω_1_ loop, the presence of testosterone imparts an increase in the amplitude of fluctuation of the amide bonds on the picosecond-nanosecond time-scale. Interestingly, this is the time-scale associated with the conformational entropy of the backbone of proteins with decreases in S^2^ scaling with an increase in conformational entropy^55^. Classically, the formation of a complex is expected to lead to a reduction in conformational entropy and an increase in S^2^. However, as discussed elsewhere, similar decreases in S^2^ (hence, increases in conformational entropy) upon complex formation have been reported before and proposed to contribute significantly to the stability of the complex by decreasing its Gibbs free energy by optimisation of its conformational entropy^56^. Accordingly, Fig. 7a displays three different and representative snapshots from the molecular dynamics simulation of the STARD6-testosterone complex in which the ligand adopts different configurations while maintaining non-polar interactions and multiple alternative H-Bonds. The distribution and the occurrence of H-bonds between STARD6 and testosterone are summarized in Fig. 7b and Supplementary Table 1. The main polar interaction between the testosterone hydroxyl group and STARD6 occurs via R71, Y81, D113 and S129; in parallel, the carbonyl group interacts mainly through S100, N191 and S129 (Fig. 7a,b and Supplementary Table 1). Altogether, those results suggest that the complex is more dynamical than the apo-form on the ps-ns timescale and that this dynamic behavior is caused by a multitude of potential configurations and intermolecular interactions between testosterone and the side-chains projected inside the STARD6 cavity with seemingly similar probability of existence or Gibbs free energy.

Overlaid to those changes in motions on the ps-ns time-scale, the binding of testosterone quenches larger scale motions in the backbone related to changes in the ^15^N chemical shifts and to amide exchange. Indeed, with the exception of a few residues (T68, G104, S105, F153, M169 and V171), μs-ms motions measured by relaxation dispersion (CPMG) are strongly damped by the presence of the ligand (Figs 4 and 6f and Supplementary Fig. 7d). The model-free analysis corroborates that the testosterone bound form appears less mobile on this time-scale (Fig. 6e). In particular, μs-ms motions in Ω_1_ loop and the N-terminal side of the α_4_ helix are completely abolished in the presence of testosterone (Fig. 6e,f and Supplementary Fig. 7d). Accordingly, the RMSF values obtained from the molecular dynamics simulations of the apo and testosterone-bound form shows a local rigidification of the Ω_1_ loop upon ligand binding (Fig. 6h and Supplementary Fig. 7e). This is further confirmed by the appearance of a resistance to the amide exchange of residues in Ω_1_ loop (F101 and A102) and the N-terminal of α_4_ helix (M188) (Fig. 6g, Supplementary Figs 6 and 7c). Moreover, such high protection factors indicates that the backbone amide of these residues must be involved in the formation of hydrogen bonds or become highly protected by their local environment in the complex. Interestingly, few residues have persistent conformational exchange at the level of their backbone.

In summary, the binding of testosterone to STARD6 causes an overall increase in flexibility on the ps-ns timescale while dampening motions of larger amplitude on the μs-ms timescale. As for the the major urinary protein, this redistribution of the conformational entropy from one timescale to another may contribute to the stabilisation of the complex by the so-called entropy-entropy compensation effect^57^,^58^. The persistence of fast-motions in the complex may also be important for the re-opening of the cavity and the release of the ligand. Indeed, such a ps-ns to μs-ms motion interplay have already been documented in the literature^49^,^50^,^51^,^56^,^57^,^58^.

## Conclusion

With the completion of the STARD6 structure presented here, the structure of all the members of the mammalian sterol binding START domains family (STARD1 and STARD4 subfamilies) is now completed. Except for some loop deletions and insertions, the structures of all these START domains are very similar; the backbone superposition of STARD1^10^, STARD3^2^, STARD4^11^, STARD5^10^ and STARD6 is shown in Supplementary Fig. 8. Their backbone superimposes onto each other with pairwise RMSDs of less than 2.0 Å (Supplementary Table 2).

We also unveil the time-scales and amplitude of the structural fluctuations proposed to be important for the binding of ligands by START domains (Fig. 7c). Most noteworthy, we demonstrate the concomitant conformational exchange in the Ω_1_ loop and the N-terminal portion of α4 in the apo-state of STARD6. We show that this leads to the coalescence of the buried binding site with the surrounding bulk water molecules to allow for ligand entry. Despite their low primary structure homologies^12^, we propose that this conformational exchange and binding mechanism, presented here, will be conserved across the fifteen human START domains. In this regard, significant fluctuations (high B-factors) in the Ω_1_ loop are conserved in the structure of the apo-forms of START domains in the majority of the crystal structures reported^2^,^10^,^11^,^14^,^59^. In addition, steered molecular dynamics simulations have been used to show that the opening of the Ω_1_ loop can play the role of a lid and allow for the exit of cholesterol in STARD1 and STARD3^15^. Also in accordance with the mechanism we propose, Baker *et al*. have engineered a disulphide bridges that links the Ω_1_ loop to the N-terminus of the α_4_ helix of STARD1. They have shown that this mutant was only functional under reducing conditions^60^. Finally, we have unveiled the presence of slightly less frequent fluctuations on the μs-ms clustered around the α_3_ helix and strands β_4_ and β_5_, lining the opposite end of the cavity (Fig. 3f, Supplementary Fig. 1c and Fig. 4a,b). High B-factors were also reported in this region of STARD11. It was suggested that these fluctuations participate to the binding and exit of ceramides^14^,^52^. Alternately, we propose that these fluctuations could be important for the expulsion of water molecules with the concomitant entry of the ligand in the binding site.

Lastly, we reported that STARD6 binds to pregnenolone, precursor of all steroid-hormones^9^. Here, we show that it also binds to testosterone with an affinity in the 10^−6 ^M range. Together, those results represent an important step towards the understanding of the biological functions of STARD6 and strongly suggest a role for STARD6 in steroid transport. Accordingly, many different *in vivo* studies realized on STARD6 point towards such a function for this protein. Indeed, STARD6 is differentially expressed in germ and brain cells during development and ageing and its nuclear localization suggests that STARD6 might play a regulatory role in cells where it is expressed^61^,^62^.

In neonatal rats brain, STARD6 is found in Purkinje cells during the first and second week after birth^63^,^64^,^65^,^66^,^67^,^68^, period that Purkinje cells are the most active in steroid synthesis^69^, when cerebellar neural circuit formation occurs^70^. In aged rats, STARD6 slightly increased in the hippocampus but diminished in the cerebrum and cerebellum cortex, suggesting a role for STARD6 during brain ageing^64^.

In adult rat testes, STARD6 is present in the seminiferous tubules and localizes in germ cell nuclei. This START protein is expressed only during the late stages of spermatogenesis, suggesting that STARD6 may play a role during cell maturation^61^,^62^. In addition, its expression in testes decreases with ageing and may be associated with decreased spermatogenesis in aged rats^61^. By analogy, in the pig ovary, STARD6 is differentially expressed during the estrous cycle, in the mid-luteal phase corpora lutea^71^.

Hopefully, the detailed structural and dynamical data obtained for steroid binding by STARD6 in this study will serve as landmarks for studying others START domain binding mechanism. Beyond the structural biology, the discovery that STARD6 binds steroids with high affinity brings new insight for studying its role in Purkinje and germ cells during development and ageing, and could eventually contribute to the resolution of certain types of pathologies occurring in the brain and those related to fertility problems.

## Additional Information

**How to cite this article**: Létourneau, D. *et al*. STARD6 on steroids: solution structure, multiple timescale backbone dynamics and ligand binding mechanism. *Sci. Rep.* **6**, 28486; doi: 10.1038/srep28486 (2016).

## Supplementary Material

Supplementary Information

## Figures and Tables

**Table 1:** Thermodynamic stability of apo-STARD6, the STARD6-testosterone complex and its apparent dissociation constant obtained from the simulation of the temperature denaturation and thermal shift analysis.

	T° (°C)	ΔH°_u_ (T°)^a^,^c^	ΔG°_u_ (25 °C)^a^	ΔG°_u_ (37 °C)^a^	K_d_^d,e^
STARD6 (apo)	54.0 (±0.3)^b^	106.5 (±2.3)^b^	9.4 (±0.5)	4.6 (±0.5)	
Testosterone	58.2 (±0.2)	117.9 (±2.3)	16.6 (±0.5)	9.5 (±0.5)	3.08 (±0.23)

^a^Values given in kcal·mol^−1^.

^b^Standard deviation of the fits.

^c^Calculated at 1:5 ratio.

^d^10^−6 ^M^−1^.

^e^at T° of apo-STARD6.

**Table 2:** Structural statistics for STARD6.

Restraints for final structure calculations	
NOE distance restraints	
Intraresidue (|i–j| = 0)	1612
Sequential (|i–j| = 1)	1237
Medium range (1 < |i–j| < 5)	732
Long range (|i–j| ≥ 5)	1308
Ambiguous NOE	214
Total NOE distance restraints	5103
Hydrogen bonds	80 × 2
Φ and ψ angles dihedral angle restraints^a^	302
**Structure statistics (20 structures)**	
Average number of distance violations per structure (>0.5 Å)	0.2 ± 0.4
Average number of dihedral angle violations per structure (>5°)	0.3 ± 0.4
RMS deviations from experimental data	
Average distance restraint violation (Å)	0.043 ± 0.001
Average dihedral restraint violation (°)	0.60 ± 0.08
RMS deviation to mean coordinates	
Backbone heavy atoms (Å)	1.18 ± 0.18
All heavy atoms (Å)	1.80 ± 0.14
For the START domain consensus sequence^b^	
Backbone heavy atoms (Å)	0.80 ± 0.10
All heavy atoms (Å)	1.39 ± 0.12
For secondary structures only	
Backbone heavy atoms (Å)	0.43 ± 0.07
All heavy atoms (Å)	0.98 ± 0.10
Ramachandran plot statistics^c^ (%)	
Residues in most favored regions	73.7
Residues in additionally allowed regions	22.8
Residues in generously allowed regions	2.9
Residues in disallowed regions	0.5

^a^Φ and ψ angles were derived from the program DANGLE.

^b^START domain consensus sequence comprise residues 1–208.

^c^Ramachandran plot statistics were generated using PROCHECK_NMR for the residues 1–208.
