# Supplementary material for: STARD6 on steroids: solution structure, multiple timescale backbone dynamics and ligand binding mechanism
Source: Sci Rep. 2016 Jun 24;6:28486. doi: 10.1038/srep28486 (PMC4919784; doi:10.1038/srep28486)
Supplement: Supplementary Information [file srep28486-s1.pdf]

SUPPLEMENTARY INFORMATION

STARD6 on steroids: solution structure, multiple timescale backbone dynamics and ligand binding mechanism.

Danny Létourneau[1-4], Mikaël Bédard[1-4], Jérôme Cabana[1-4], Andrée Lefebvre[1], Jean-Guy LeHoux[1] & Pierre Lavigne[1-4]

[1]Département de Biochimie, Faculté de Médecine et des Sciences de la Santé, Université de Sherbrooke, Sherbrooke, Qc., Canada

[2]Institut de Pharmacologie de Sherbrooke, Université de Sherbrooke, Sherbrooke, Canada

[3]PROTEO; Regroupement Stratégique sur la Fonction, la Structure et l'Ingénierie des Protéines, Université Laval, Québec, Qc., Canada

[4]GRASP; Groupe de Recherche Axé sur la Structure des Protéines, McGill University, Montréal, Qc. Canada

Correspondence should be addressed to P.L. and J.-G.L. (pierre.lavigne@usherbrooke.ca; jean-guy.lehoux@sherbrooke.ca)

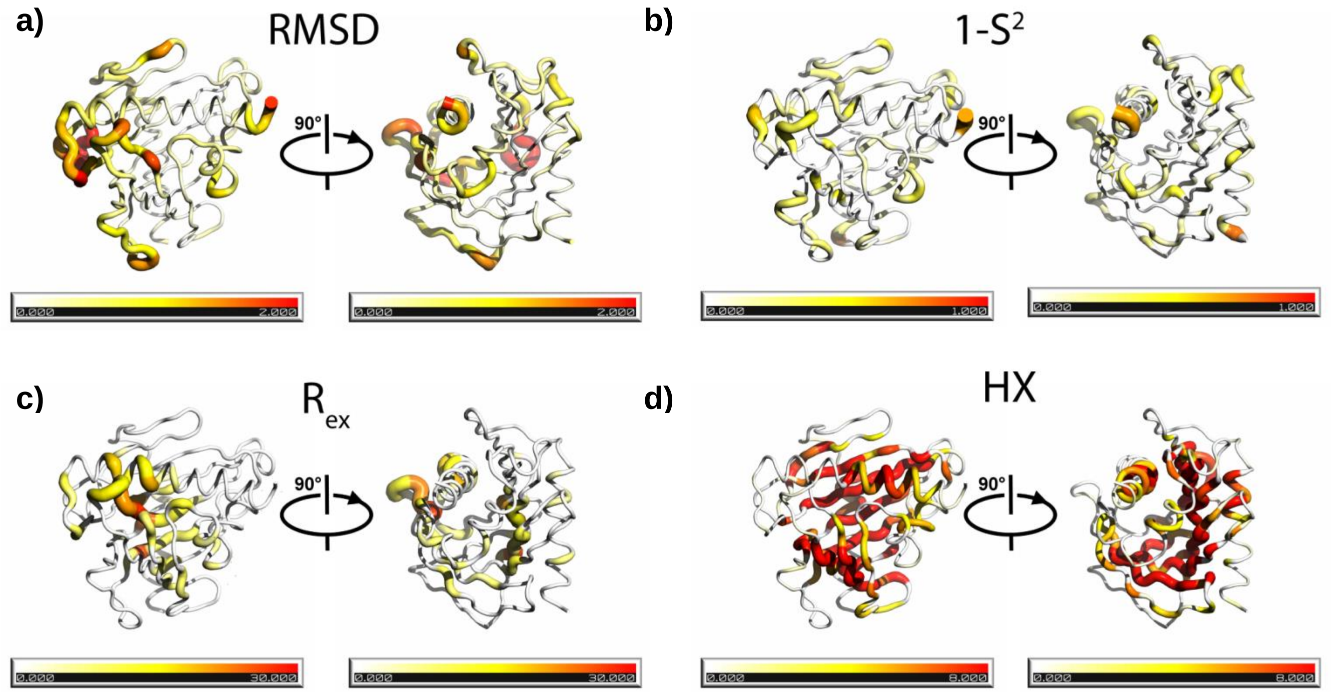

Supplementary Figure 1: Apo-STARD6 dynamic parameters mapped onto the most representative STARD6 conformer (lowest energy). (**a**) Mean backbone RMSD calculated from the ensemble of the 20 lowest energy conformers. (**b**) 1-S^2 (squared order parameter S^2). (**c**) R_{ex} determined by the relaxation dispersion method (CPMG). (**d**) H^N protection factors (P_f). Worm radii are proportional to the parameters mapped and coloured in a white to red gradient; lower values (white) have thinner backbone worms, higher values (red) have thicker backbone worms and intermediate values are yellow.

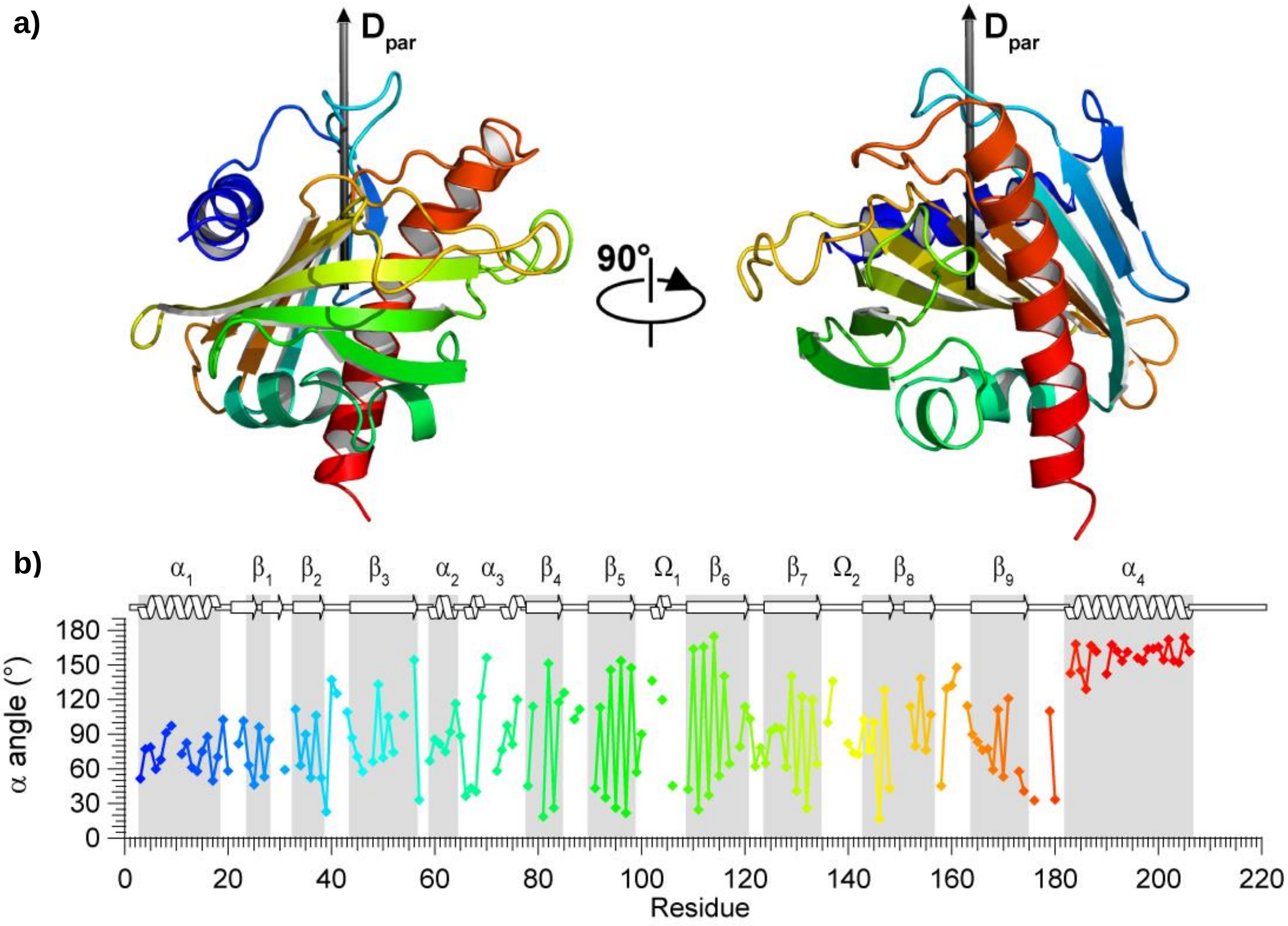

Supplementary Figure 2: The axially symmetric rotational diffusion of STARD6. (**a**) The structure of STARD6 in the diffusion frame depicting the unique axis of the diffusion tensor, D_{par}. (**b**) Direction cosines or α angles of the NH vectors of each residue (except prolines) relative to the unique axis of the diffusion tensor.

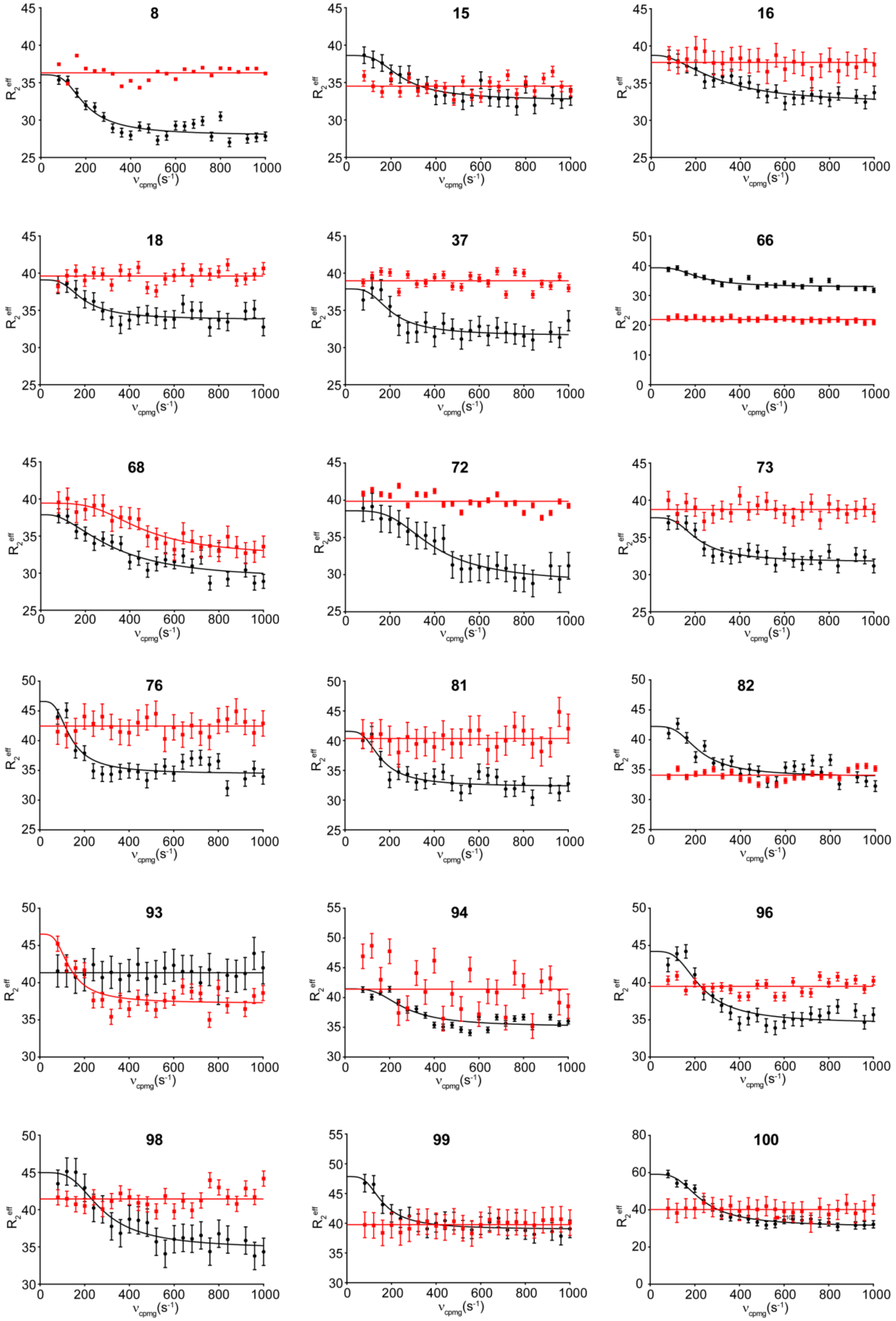

8
15
16
18
37
66
68
72
73
76
81
82
93
94
96
98
99
100
R_2^{eff}
$\nu_{cpmg}(s^{-1})$

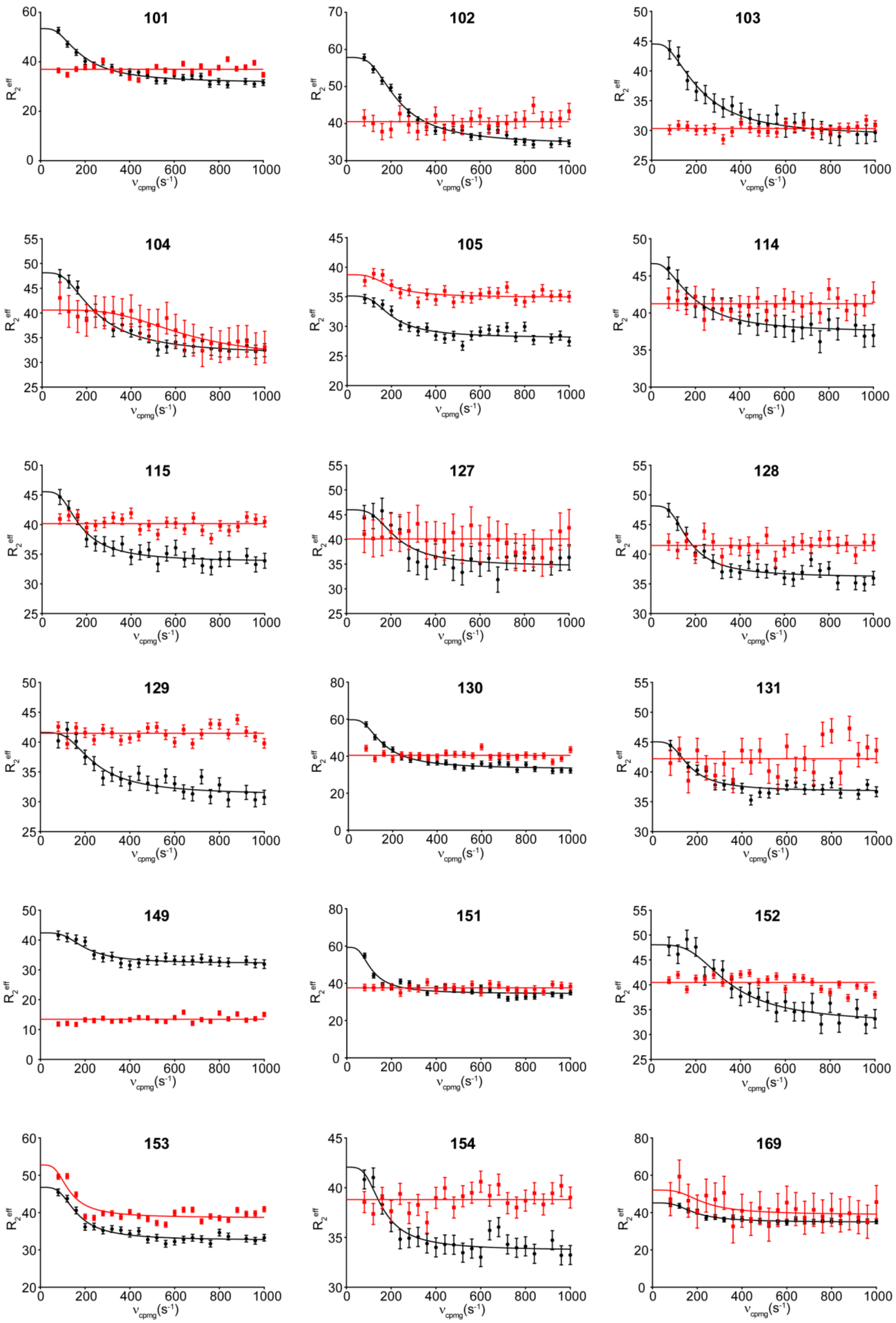
101
102
103
104
105
114
115
127
128
129
130
131
149
151
152
153
154
169
R_2^{eff}
ν_{cpmg} (s^{-1})

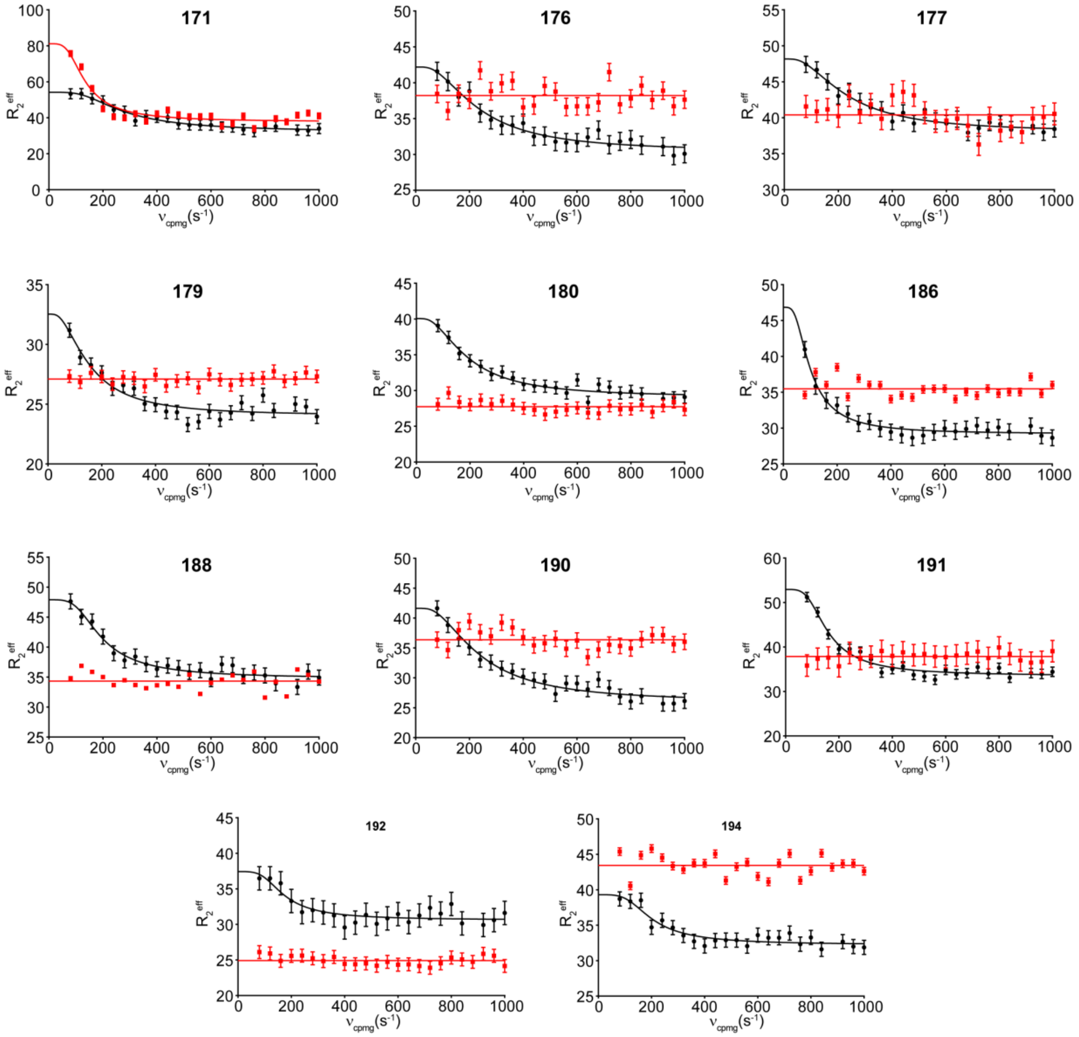

Supplementary Figure 3: ^{15}N relaxation dispersion profiles for STARD6 non-overlapping cross peaks, recorded at a static magnetic field strengths of 600 MHz at 25°C and in the absence (black) and presence of testosterone (red). Error bars represent uncertainties in relaxation rates. CPMG field strengths, ν_{cpmg}, ranged from 80 to 1000 Hz and the constant relaxation delay was 50 ms. Spectra were collected as series of two-dimensional data sets. Duplicate data sets were recorded at selected ν_{cpmg} values for error analysis. Peak intensities observed in the ^{1}H-^{15}N HSQC spectra were converted into effective relaxation rates ($R_{2,eff}$) and uncertainties in relaxation rates were calculated from repeat experiments. $R_{2,eff}$ were fitted as described in the materials and methods section.

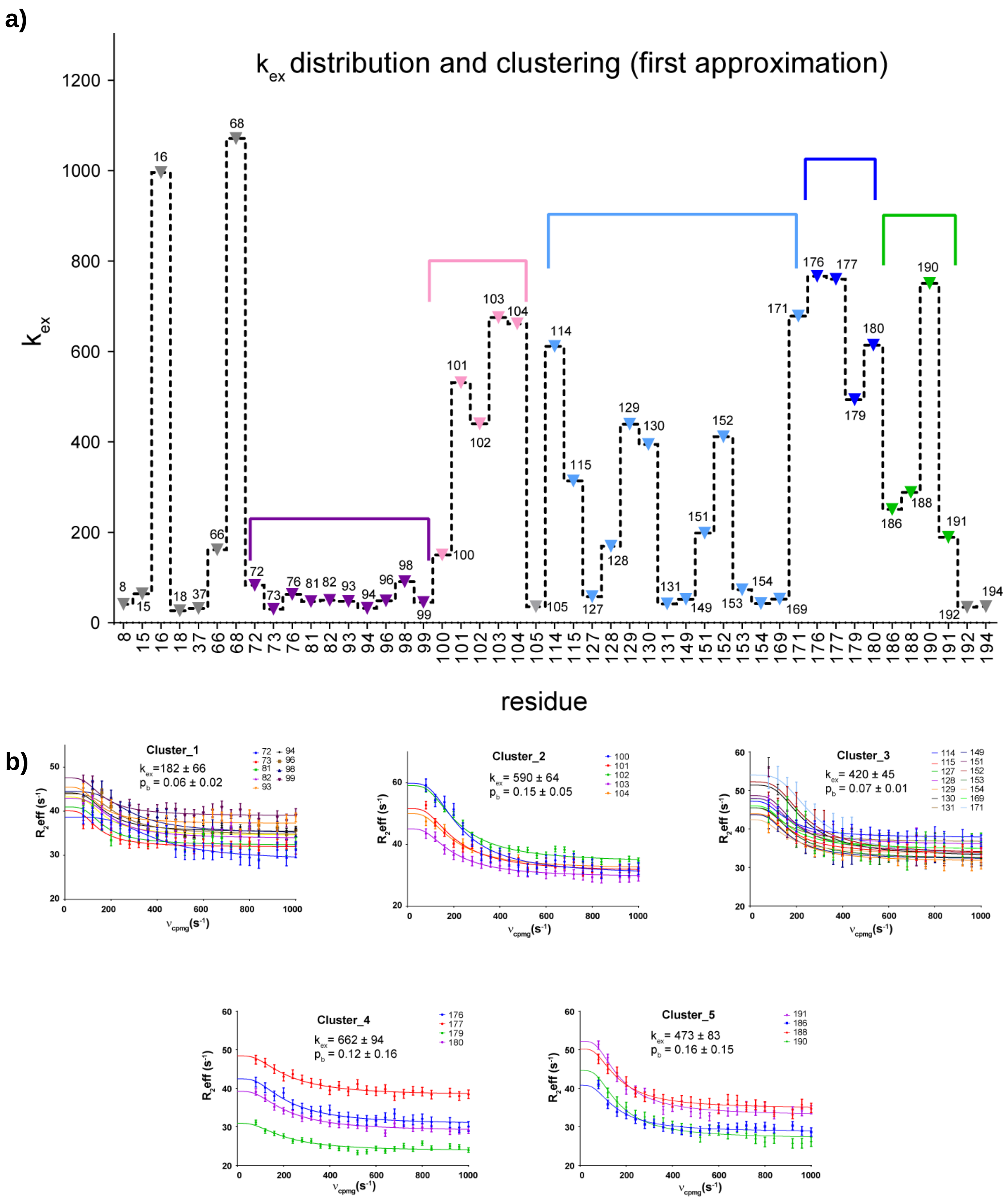

Supplementary Figure 4: Cluster analysis based on initial k_{ex} values obtained, secondary structure elements and inter-residue secondary or tertiary connectivity. (a) Distribution of the individual and initial k_{ex} values exchange rate constant (k_{ex}) and clustering analysis. Parameters k_{ex} and p_b were shared parameters for each cluster, and R_2^0, Dω were individually determined. Residues presenting cross peak overlap in both the apo-STARD6 and the testosterone bound form were discarded from the relaxation dispersion analysis. Residues for which we were not able to cluster based on first k_{ex} approximation were not included in the cluster analysis (Grey). Cluster

1 (Purple: involving residues in the α_3 helix and β_4 strand), cluster 2 (pink: involving the Ω_1 loop), cluster 3 (cyan: involving the bottom of the β-sheet), cluster 4 (blue: involving the N-terminal end of α_4 helix) and cluster 5 (green: involving the α_4 helix). (**b**) ^{15}N relaxation dispersion profiles for different clusters. Parameters k_{ex} and p_b were shared parameters for each cluster, and R_2^0, Dω were individually determined. Residues presenting crosspeak overlap were discarded from the relaxation dispersion analysis.

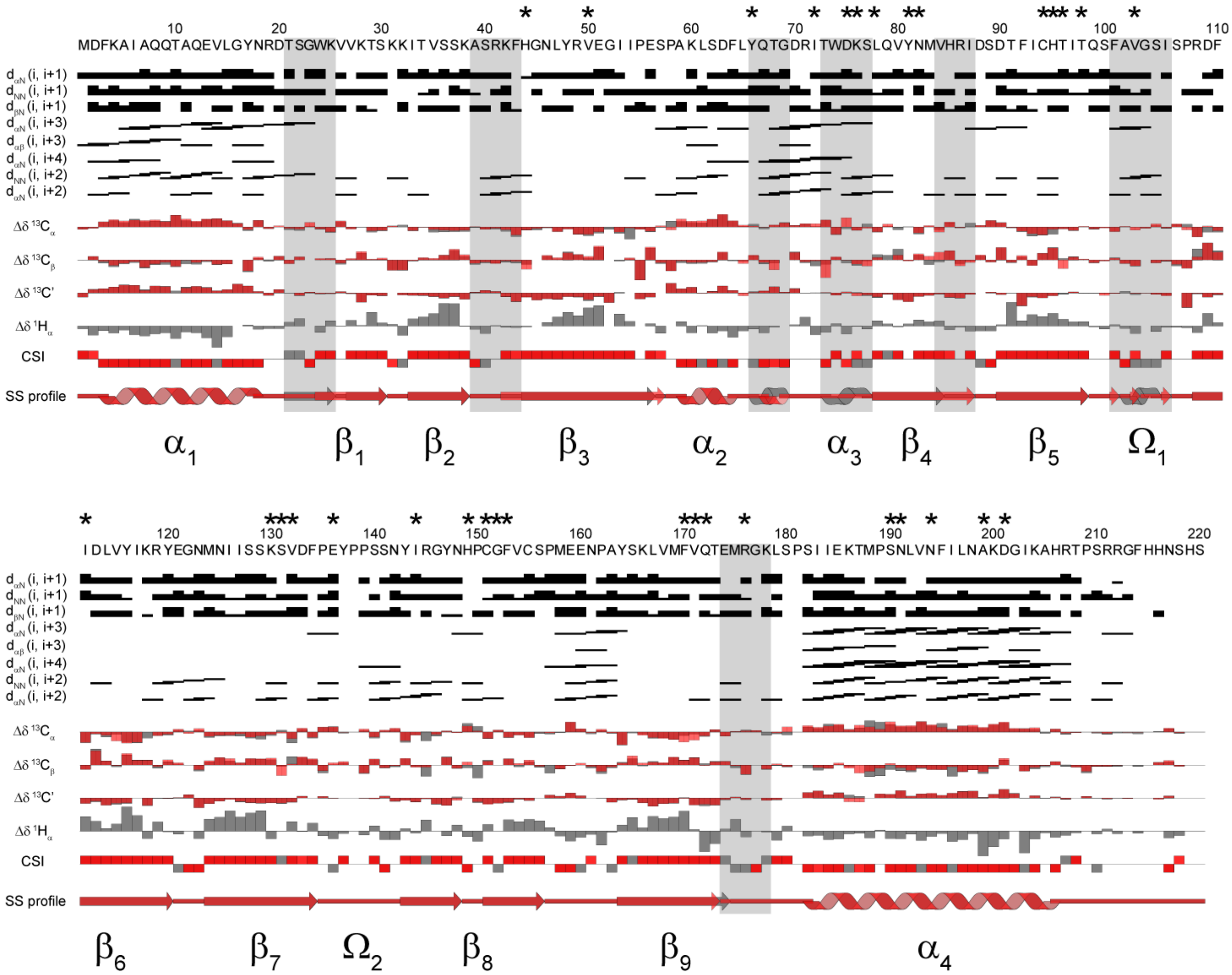

Supplementary Figure 5: Sequence specific secondary structure for STARD6. Secondary chemical shifts of $^{13}C^{\alpha}$, $^{13}C^{\beta}$, $^{13}C'$, $^{1}H^{\alpha}$ and CSI (chemical shift index) and secondary structure profile from CSI values for apo-STARD6 (black) and testosterone bound (red). α-Helices and β-strands are identified by consecutive CSI values of − 1 and 1, respectively. One letter abbreviations correspond to the amino acid sequence. The apo-STARD6 NOE connectivities support the presence of the predicted secondary structures. The upper set of rows illustrate the sequential (i, i+1) and medium range (i, i+2, i, i+3 and i, i+4) NOE and are indicated by the lines connecting the two coupled residues. The relative NOE intensities are indicated by the thickness of the bars. Residues presenting CSDs over the mean values are denoted by black stars.

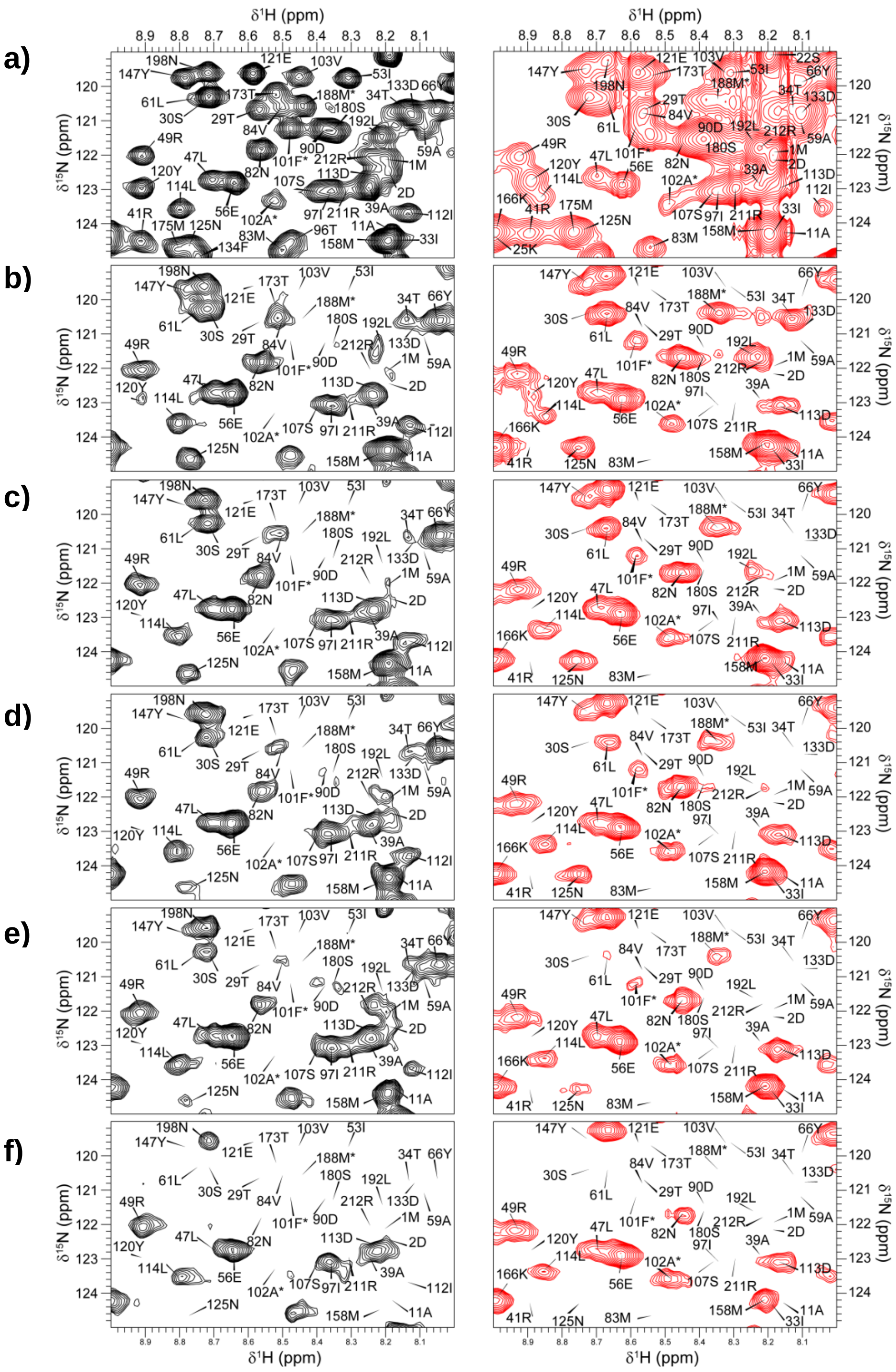

Supplementary Figure 6: ^{1}H-^{15}N-HSQC spectra of apo-STARD6 (black/left) and STARD6-testosterone complex (red/right) recorded along the H/D exchange experiments. Before exchange (**a**) 30 min (**b**), 3 h (**c**), 6 h (**d**) 12 h (**e**) and (**f**) 24 h after solubilisation of the lyophilized protein in D_2O at 25 ºC. Assignments are as indicated. Black stars denote residues in Ω_1 loop (F101 and A102) and the N-terminal of α_4 helix (M188) that become resistant to the amide exchange upon binding of testosterone.

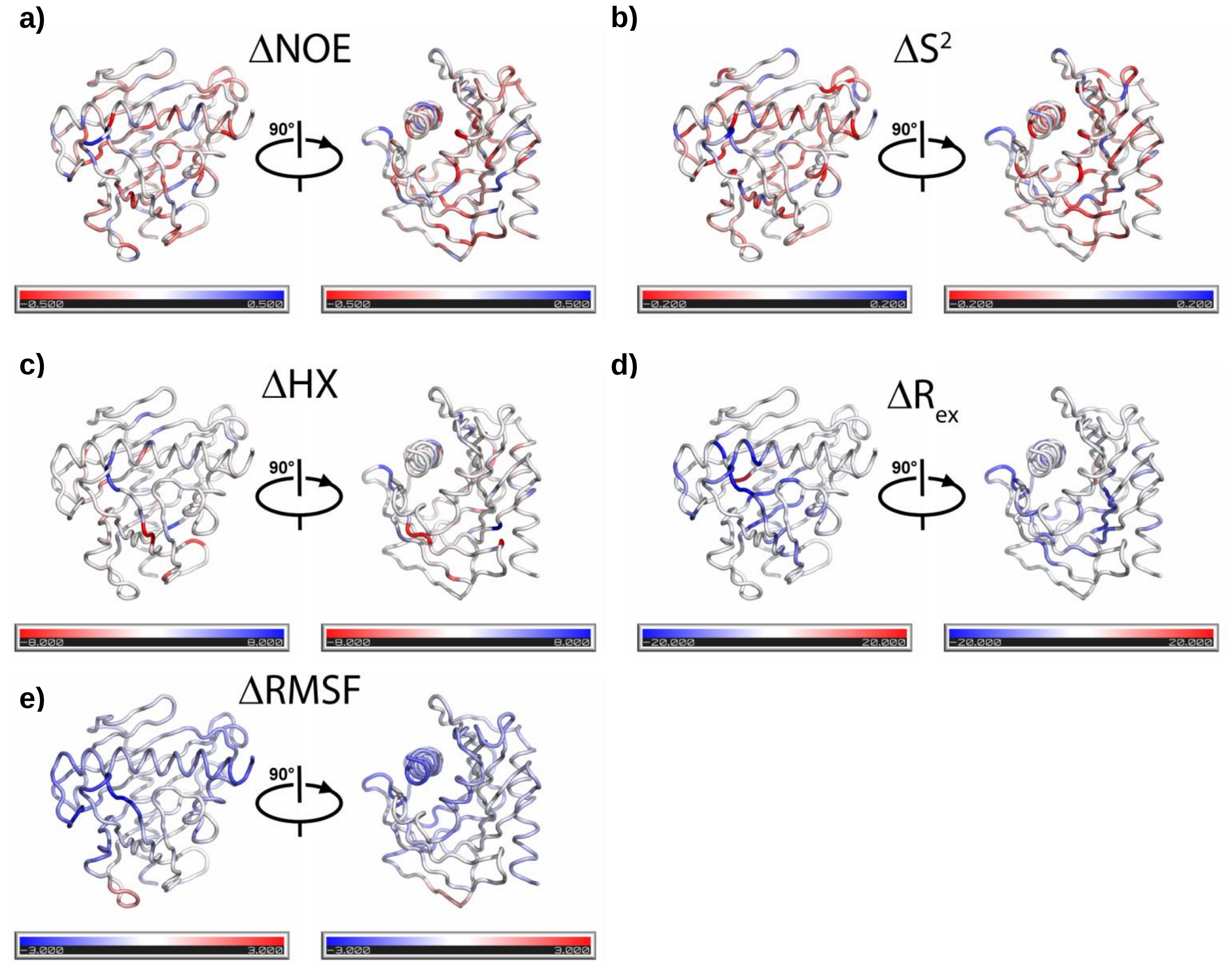

Supplementary Figure 7: Changes in the dynamics of the backbone of STARD6 upon testosterone binding mapped onto the apo-structure. (**a**) NOE differences (complex - apo). (**b**) S^2 differences mapped onto the STARD6 structure. (**c**) Changes in H^N protection factors (complex – apo). (**d**) Changes in R_{ex} (complex – apo). (**e**) RMSF differences (complex-apo). Colored according to the scales indicated. Residues with loss of mobility or becoming more protected (HX) are in blue, while those showing an increase in flexibility or less protected from the solvent are colored red.

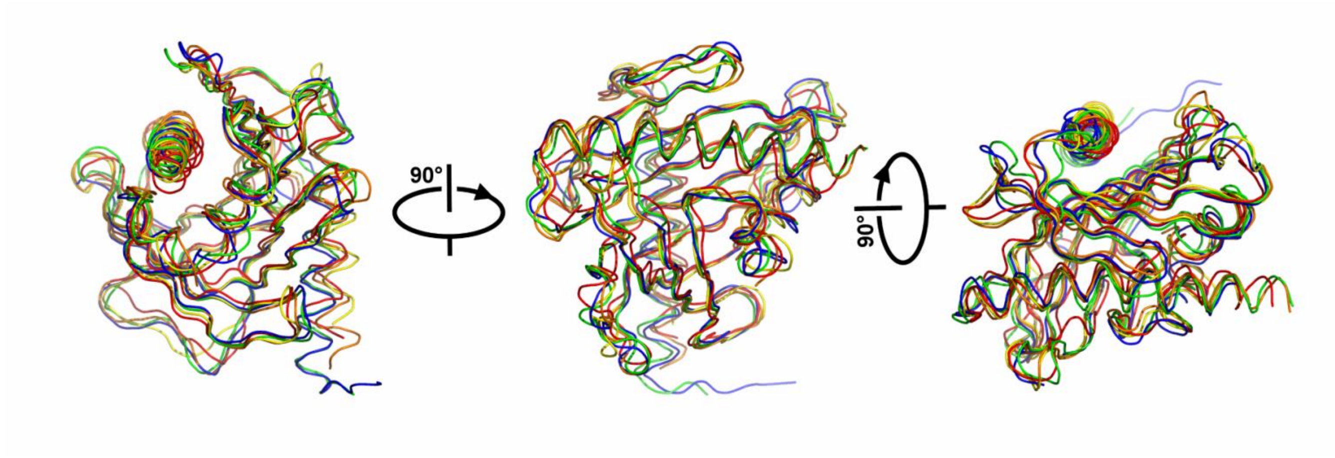

Supplementary Figure 8: Superposition of STARD1 and STARD4 sub-families members onto STARD6 (2MOU). Orthogonal views of the superposition of the START domain structures of STARD1 and STARD4 subfamilies (blue, STARD1 (3P0L_A); green, STARD3 (1EM2_A); yellow, STARD4 (1JSS_A); orange, STARD5 (2R55_A); red , STARD6 (2MOU).

Supplementary Table 1. Distribution and occurrence of H-bonds between STARD6 and testosterone polar groups.

D6 residue	Avg. H-bonds per frame	Testosterone polar groups
R71	0.321	OH
S129	0.246	OH and CO
D113	0.229	OH
Y81	0.174	OH
N191	0.141	CO
S100	0.124	CO
L65 (CO)	0.119	OH
Q67	0.103	OH
N198	0.095	OH
R109	0.046	CO
N148	0.030	OH and CO
D70 (CO)	0.015	OH
T173	0.015	CO
T187	0.012	CO
S77	0.007	CO
T96	0.001	OH

Supplementary Table 2. Superposition of STARD1 and STARD4 sub-families members to STARD6 (2MOU).

Protein name (PDB ID code)	CEalign (1) RMSD (Å) (number of aligned residues)	Pymol default alignment RMSD (Å) (number of aligned residues)	% I.D.
STARD1 (3P0L)	1.96 (192)	1.49 (154)	22.7
STARD3 (1EM2)	2.01 (200)	1.86 (148)	19.5
STARD4 (1JSS)	1.90 (192)	1.28 (171)	28.4
STARD5 (2R55)	1.69 (200)	1.49 (187)	33.8

1. Shindyalov IN & Bourne PE (1998) Protein structure alignment by incremental combinatorial extension (CE) of the optimal path. *Protein engineering* 11(9):739-747.
